# Cell-Penetrable Peptide-Conjugated FADD Induces Apoptosis and Regulates Inflammatory Signaling in Cancer Cells

**DOI:** 10.3390/ijms21186890

**Published:** 2020-09-19

**Authors:** Kishu Ranjan, Bhargav N Waghela, Foram U Vaidya, Chandramani Pathak

**Affiliations:** Department of Cell Biology, School of Biological Sciences & Biotechnology, Indian Institute of Advanced Research, Koba Institutional Area, Gandhinagar 382 007, Gujarat, India; kishu.ranjan@yale.edu (K.R.); wbhargav@gmail.com (B.N.W.); foram.vaidya11@gmail.com (F.U.V.)

**Keywords:** cancer, apoptosis, FADD, peptides, NF-κB, inflammation

## Abstract

Dysregulated expression of Fas-associated death domain (FADD) is associated with the impediment of various cellular pathways, including apoptosis and inflammation. The adequate cytosolic expression of FADD is critical to the regulation of cancer cell proliferation. Importantly, cancer cells devise mechanisms to suppress FADD expression and, in turn, escape from apoptosis signaling. Formulating strategies, for direct delivery of FADD proteins into cancer cells in a controlled manner, may represent a promising therapeutic approach in cancer therapy. We chemically conjugated purified FADD protein with cell permeable TAT (transactivator of transcription) peptide, to deliver in cancer cells. TAT-conjugated FADD protein internalized through the caveolar pathway of endocytosis and retained in the cytosol to augment cell death. Inside cancer cells, TAT-FADD rapidly constituted DISC (death inducing signaling complex) assembly, which in turn, instigate apoptosis signaling. The apoptotic competency of TAT-FADD showed comparable outcomes with the conventional apoptosis inducers. Notably, TAT-FADD mitigates constitutive NF-κB activation and associated downstream anti-apoptotic genes *Bcl2*, *cFLIP_L_*, *RIP1*, and *cIAP2*, independent of pro-cancerous TNF-α priming. In cancer cells, TAT-FADD suppresses the canonical NLRP3 inflammasome priming and restricts the processing and secretion of proinflammatory IL-1β. Our results demonstrate that TAT-mediated intracellular delivery of FADD protein can potentially recite apoptosis signaling with simultaneous regulation of anti-apoptotic and proinflammatory NF-κB signaling activation in cancer cells.

## 1. Introduction

The Fas-associated death domain (FADD) protein orchestrates several cellular pathways, including apoptosis, cell cycle regulation, autophagy, and inflammation [1,2]. The molecular structure of FADD consists of the N-terminal death effector domain (DED) and C-terminal death domain (DD). Upon activation of death receptors (DRs), the DD of FADD interacts with the DD of DRs, and facilitates the DED of FADD to oligomerize with DED containing pro-apoptotic proteins, such as procaspase-8/10, to form a multimeric death-inducing signaling complex (DISC) [3,4]. Indeed, the constituted DISC assembly concomitantly activates the canonical apoptosis signaling [5,6]. In cancer cells, the DED-containing protein cFLIP_L_ (cellular FLICE inhibitory protein) competitively exclude caspase-8 binding to FADD at DISC and inhibits apoptosis signaling [7,8]. Importantly, post-translational modifications (PTMs) and nuclear localization of FADD have been reported in cancer cells, which further challenge the pro-apoptotic competency of FADD to instigate apoptosis signaling [9,10,11]. In this context, earlier studies demonstrated that low expression of FADD in cancer cells further exacerbates the severity of disease [12,13,14,15]. Moreover, somatic mutation in FADD [16] and elevated expression of cFLIP [17] has been attributed in the pathogenesis of colon carcinoma. We previously reported that induced expression of FADD regulates cFLIP_L_ expression and favors procaspase-8 binding to DISC, independent of DR stimulation [18,19,20]. Targeting FADD as a therapeutic candidate would be a promising approach to reinstate apoptosis signaling in cancer cells.

It is well established that the tumor microenvironment is enriched with various pro-tumorigenic stimuli, such as tumor necrosis factor-α (TNF-α), inflammatory cytokines, and growth factors, which constitutively activates NF-κB signaling and cell proliferation [21,22,23]. Noteworthy, a constitutive NF-κB activation, leads to aberrant transcriptional priming of anti-apoptotic genes, such as *Bcl-2*, *TRAF2*, *cFLIP*, *cIAPs*, and *RIP1* which further abrogates apoptosis signaling [22,24]. On the other side, pathogen recognition receptor (PRR)-mediated NF-κB activation induces transcriptional priming of proinflammatory genes to maintain the tumor microenvironment [25,26]. In this context, the NLRP3 inflammasome complex consisting of NLRP3 (nucleotide-binding domain leucine-rich repeat (NLR) and pyrin domain containing receptor 3), ASC (Apoptosis-associated speck-like protein containing a CARD), and pro-caspase-1 protein facilitates the proteolytic processing of NF-κB-induced proinflammatoryIL-1β to promote tumor growth [27,28,29]. We and others previously reported that transient expression of FADD abrogates NF-κB activation [18,30]. A recent report revealed that canonical NLRP3 inflammasome activation induces the secretion of soluble FADD protein in human monocytes and macrophages [31]; however, the exact molecular mechanism of FADD, in the regulation of NLRP3 inflammasome signaling is less explored in the context of apoptosis.

Given the importance of FADD in apoptosis and pro-tumorigenic NF-κB signaling, FADD protein may demonstrate a tremendous potential to mitigate cancer progression. In this context, few studies investigated the vector-based FADD gene therapy approach in regulation of tumor growth [32,33,34] and apoptosis in synoviocytes [35]; however, adenoviral or vector-based approaches have limited control over protein expression and host-derived factors. Other novel ways to directly deliver FADD protein in cancer cells are less explored. Recent advancement of direct protein delivery to cells provides a novel way to enrich poorly expressed proteins to the intracellular compartments [36,37,38]. The recent developments in therapeutic applications of small cell-penetrating peptides (CPPs), such as TAT (trans-acting activator of transcription) peptides, have been successfully validated to transport macromolecules, such as nucleic acids and proteins, across the cell membrane [39,40]. In this study, we chemically conjugated human FADD protein with TAT peptide for delivery into cancer cells and investigated the potential of the TAT conjugate FADD (TAT-FADD) in the regulation of apoptosis and NF-κB signaling. Our results showed that the TAT-FADD conjugate efficiently internalizes across the cells and forms a DISC assembly, followed by augmentation of apoptotic signaling in cancer cells. Moreover, TAT-FADD targets NF-κB signaling to suppress the expression of anti-apoptotic genes *Bcl2*, *cIAPs*, *RIP1*, and *cFLIP_L_* in cancer cells, and TAT-FADD abolishes NLRP3 inflammasome transcriptional priming and processing of IL-1β in colon carcinoma HCT116 cells. This study provides a novel insight of the molecular mechanism and delivery of FADD protein, which can target cancer cell proliferation and NF-κB activation with subsequent suppression of pro-tumorigenic and proinflammatory signaling. This indispensable approach may be beneficial to the consideration of protein as a drug candidate in the revolution of anticancer therapy.

## 2. Results

### 2.1. Conjugation and Characterization of TAT-FADD

**Figure 1:**
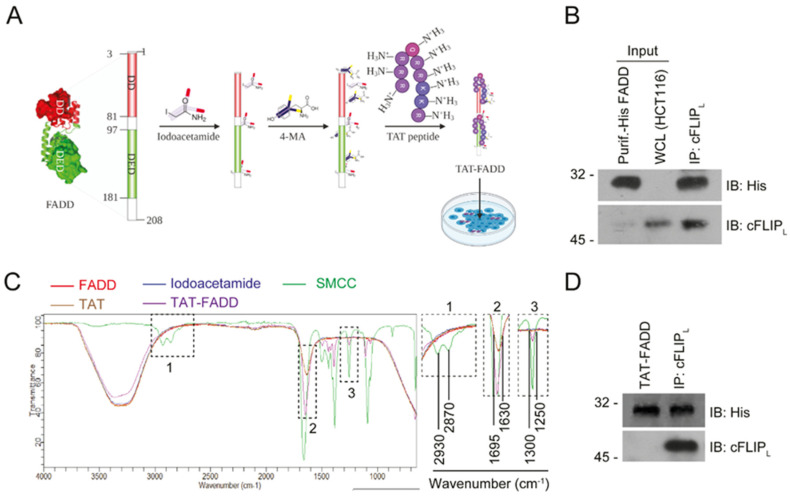
Human FADD protein conjugated with TAT peptide. (**A**) Schematic diagram showing the chemical conjugation of FADD protein (PDB ID-2GF5) with linker iodoacetamide and SMCC (4-MA) followed by TAT peptide. (**B**) In vitro protein interaction with purified His-tagged FADD and whole cell lysate (WCL) from HCT 116 cells, the immunoprecipitated (IP’ed) His-FADD interacts with binding partner cFLIP_L_ protein, input lanes represent loading controls, as assessed by Western blot, molecular weight marker left to each blot. (**C**) The FT-IR analysis of TAT-FADD conjugate; inset box 1, 2, and 3 represent the corresponding peaks from the representative FT-IR spectrum. (**D**) In vitro protein interaction with His-taged TAT-FADD conjugate and whole cell lysate (WCL) from HCT 116 cells, the IP’ed His (TAT-FADD) interacts with cFLIP_L_ protein, as assessed by Western blot, molecular weight marker left to each blot.

The schematic representation of human FADD protein conjugated with TAT peptide is shown in Figure 1A. We purified recombinant His-tagged human FADD protein through the guanidine-HCl (Gn-HCl) denaturation method and confirmed the purity of the protein through SDS-PAGE staining (Figure S1A). The biophysical characterization of purified FADD through mass spectrometry demonstrated and expected m/z peak at 2955.609 Da (Figure S1B). To examine the integrity of FADD protein-binding epitopes, we conducted an in vitro protein interaction assay, and found that purified FADD protein efficiently interacts with its cognate partner cFLIP_L_ (Figure 1B). Next, we conducted an in vitro conjugation of purified FADD protein with TAT peptides in the presence of linkers SMCC (Succinimidyl- trans-4-(N-maleimidylmethyl)cyclohexane-1-carboxylate) and iodoacetamide, and analyzed the conjugates through FT-IR analysis. The FT-IR spectrum of TAT-FADD conjugate showed that two broad peaks of acidic hydroxyl in the wavelength range of 2870–2930 cm^−1^ (inset box 1), acidic amide in the wavelength range of 1630–1695 cm^−1^ (inset box 2), and an aromatic ester peak in the wavelength range of 1300–1250 cm^−1^ (inset box 3) were reduced in the TAT-FADD conjugate (purple line) as compared to the linker SMCC (MA, green line), which confirmed that theses linker bonds of SMCC cross-react with FADD protein SH- groups to stabilize the structure of protein (Figure 1C). We next examined the protein quality and binding integrity of TAT-FADD conjugate. We found that TAT-FADD retained the molecular, as confirmed in SDS-PAGE gel (Figure S1C). Moreover, an in vitro protein interaction assay confirmed that TAT-FADD conjugate efficiently interacted with its canonical partner cFLIP_L_ (Figure 1D). Altogether, these results demonstrate that purified FADD protein successfully conjugated with TAT-peptide and retained the functional binding integrity.

### 2.2. TAT-FADD Efficiently Internalized in the Cells and Retained in the Cytosol

**Figure 2:**
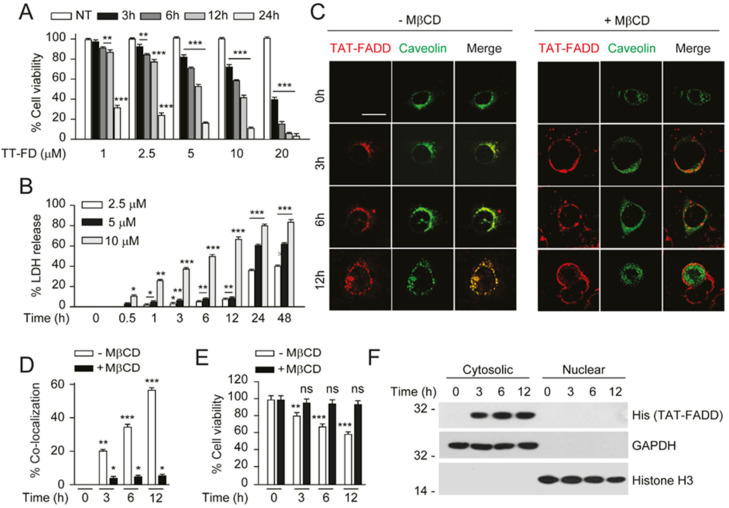
TAT-FADD efficiently delivered inside the cells and retained in the cytoplasm. (**A**,**B**) HCT 116 cells were treated with TAT-FADD (TT-FD) at the mentioned concentrations for given time points and, (**A**) analysis of cell viability and (**B**) percent LDH release. (**C**–**E**) HCT 116 cells were transfected with GFP-Caveolin1 for 24 h followed by pre-incubation with MβCD (+MβCD; right panel) for 4 h, further cells were left untreated (0 h) or treated with 5 µM of TAT-FADD for 3–12 h, (**C**) the internalized TAT-FADD was immunostained with anti-His antibody (His tagged FADD) followed by counterstaining with DAPI and analyzed by confocal microscopy, representative of 25 cells from 3 different fields; scale bar 10 µm and (**D**) the % localization of TAT-FADD with GFP-Caveolin1 from ‘C’ was calculated with Image J software, (**E**) analysis of cell viability in the absence (−) and presence (+) of MβCD. (**F**) HCT 116 cells were treated with 5 µM of TAT-FADD for 3–12 h followed by analysis of cytosolic and nuclear fractions; GAPDH and histone H3 was used as cytosolic and nuclear protein markers, respectively, molecular weight marker left to each blot. The 0 h represents untreated cells. In (**A**), significance compared between non-treated (NT) and TAT-FADD treated cells; in (**B**), significance compared between non-treated (0 h) and TAT-FADD treated cells; in (**D**,**E**), significance is compared between unprimed (-MβCD; white bars) and primed (+MβCD; black bars) cells treated with TAT-FADD. h, hours; ns, non-significant. Mean  ±  SD; * *p*  ≤  0.05, ** *p*  ≤  0.01, and *** *p*  ≤  0.001.

In order to obtain the optimal time and concentration-dependent intracellular uptake and signaling of TAT-FADD, we first treated the colon carcinoma HCT116 cells with 1, 2.5, 5, 10, and 20 µM of TAT-FADD conjugate for various time points. We found that 2.5, 5, 10, and 20 µM of TAT-FADD treatment gradually reduced the viability of cells, and 5, 10, and 20 µM of TAT-FADD exhibited higher significance in reducing cell viability and apoptotic death; the 5 and 10 µM treatment showed a gradual effect compared to the 20 µM treatment (Figure 2A; Figure S2A,B). Next, we investigated the cellular toxicity in response to 2.5, 5, and 10 µM of TAT-FADD conjugate. We measured the release of cellular LDH (a marker of necrotic cell death), and found that the 10 µM treatment exhibited higher toxicity to cells, as compared to 2.5 and 5 µM of TAT-FADD (Figure 2B). Based on the cell viability and toxicity results, we selected the 5 µM TAT-FADD conjugate treatment for the cells in the remaining studies (unless otherwise stated). Next, we examined the effect of 5 µM TAT-FADD on the viability of different origins of cancer and transformed cell lines. We found 5 µM TAT-FADD effectively reduced the viability of cancer cell lines MCF-7, HeLa, HepG2, and transformed lines HEK293 and RAW264.7 with the progression of time (Figure S2C). Moreover, we confirmed that the chemical cross linkers (SMCC and iodoacetamide), unconjugated FADD protein, and TAT peptide had no toxic effect on cells (Figure S2D,E). We next investigated the mechanism through which TAT-FADD internalized across the cells. Previous studies reported that TAT peptid- conjugated cargoes penetrate across the membrane through the caveolin-mediated endocytic pathway [41,42]. We transiently expressed GFP-caveolin-1 in HCT 116 and MCF-7 cells, and treated them with TAT-FADD conjugate. We found that TAT-FADD conjugate co-localized with caveolin1 at the periphery of the membrane and efficiently endocytosed across the cells with the progression of time (Figure 2B,D; Figure S2F,G). In order to confirm whether TAT-FADD internalized through caveolin, we treated caveolin-1-transfected cells with the pharmacological inhibitor methyl-β-cyclodextrin (MβCD). We found that TAT-FADD failed to internalize across the membrane (Figure 2C,D; Figure S2F,G). Moreover, MβCD-mediated blocking of TAT-FADD cellular entry also protected the viability of cells (Figure 2E; Figure S2H). We next sought to determine the intracellular retention of TAT-FADD, post internalization in HCT116 cells. Our sub-cellular fractionation assay data confirmed that internalized TAT-FADD was retained in the cytosol till 12 h of treatment and did not translocate to the nucleus. Altogether, these results suggest that 5 µM TAT-FADD efficiently internalized across the membrane and was retained in the cytosol of HCT116 cells.

### 2.3. TAT-FADD Constitute DISC Assembly and Instigates Cell Death via Apoptosis Signaling

**Figure 3:**
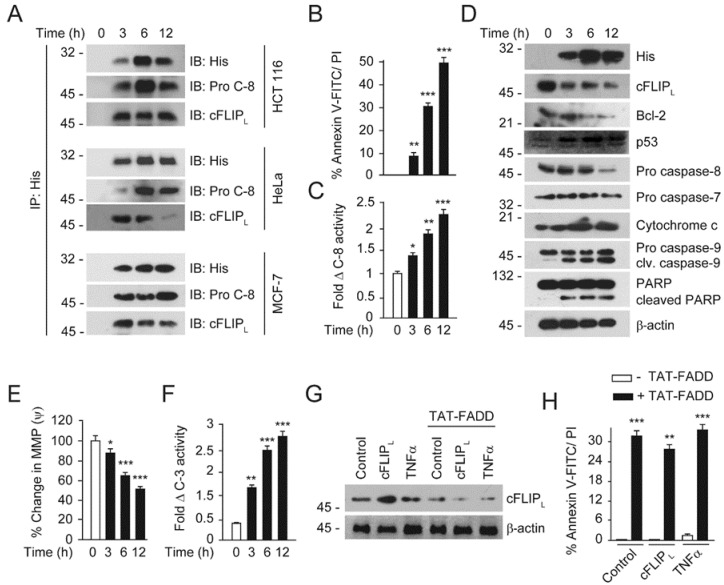
TAT-FADD constituted DISC assembly and induces apoptosis signaling. (**A**) HCT 116, HeLa, and MCF-7 cells were treated with 5 µM of TAT-FADD for 3–12 h, the His-tagged TAT-FADD was immunoprecipated (IP’ed) using anti-His antibody followed by analysis of DISC assembly proteins procaspase-8 (Pro-C-8) and cFLIP_L_, molecular weight marker left to each blot. (**B**–**F**) HCT 116 cells were treated with 5 µM of TAT-FADD for 3–12 h followed by analysis of (**B**) % apoptotic death by flow cytometry, (**C**) measurement of caspase-8 (C-8) activity, (**D**) analysis of apoptosis regulatory proteins, molecular weight marker left to each blot, (**E**) % change in MMP and (**F**) measurement of caspase-3 activity. (**G**,**H**) HCT 116 cells were transfected with pcDNA3-cFLIPL for 48 h (lane 2 and 5), primed with TNF-α (10 ng/mL) for 12 h (lane 3 and 6) followed by treatment with 5 µM of TAT-FADD for 6 h (lane 4, 5 & 6), (**G**) analysis of cFLIP_L_ expression by Western blot, molecular weight marker left to each blot and (**H**) % apoptotic death by a Tali™ image-based cytometer; control represents vector transfected and non-TNF-α-primed cells. In (**B**,**C**,**E**,**F**), significance compared between non-treated (represents as 0 h) and TAT-FADD-treated cells; in H, significance compared between non-treated (white bars) and TAT-FADD-treated cells (black bars); control in white bar represents a vector transfected and non-TNF-α-primed cells. h, hours; clv, cleaved; MMP, mitochondrial membrane potential. Mean  ±  SD; * *p*  ≤  0.05, ** *p*  ≤  0.01 and *** *p*  ≤  0.001.

We next investigated whether post internalization cytosolic TAT-FADD assembles DISC inside the cells. We treated cancer cell lines HCT 116, HeLa, and MCF-7, and a transformed cell line HEK293 with TAT-FADD and examined the constitution of DISC assembly. We found that within 3 h of treatment, TAT-FADD efficiently recruit procaspase-8 and assembled DISC with the progression of time. In contrast, we found significantly reduced binding of the anti-apoptotic protein cFLIP_L_ to DISC (Figure 3A; Figure S3A). We next examined the apoptotic potential of TAT-FADD in HCT 116 cells. We found that TAT-FADD treatment augments apoptotic death in HCT 116 with the progression of time, compared to untreated cells (Figure 3B, Figure S3B). Moreover, TAT-FADD significantly induces caspase-8 activity and targets the expression of anti-apoptotic protein cFLIP_L_ and Bcl-2, with subsequent activation of pro-apoptotic cytochrome c, pro-caspase-7, pro-caspase-9, and PARP (Poly (ADP-ribose) polymerase) (Figure 3C,D). Importantly, TAT-FADD treatment progressively induces p53 expression in HCT116 cells (Figure 3D). We further confirmed that in MCF-7 and HeLa cells, treatment with TAT-FADD regulates the expression of anti-apoptotic protein cFLIP_L_ (Figure S3C,D). We found a significant loss of mitochondrial membrane potential with concurrent induction in caspase-3 activity in TAT-FADD-treated cells (Figure 3E,F). To corroborate whether TAT-FADD specifically orchestrates caspase-mediated apoptosis signaling, we blocked caspase activation through the chemical inhibitor zVAD. Interestingly, inhibiting caspase activation significantly blocked TAT-FADD-mediated apoptosis cell death (Figure S3E–G). In an alternate approach, we transiently induced the expression of anti-apoptotic protein cFLIP_L_ by overexpression and TNF-α priming in HCT116, followed by treatment with TAT-FADD. We found that TAT-FADD significantly reduces the expression of anti-apoptotic cFLIP_L_, with concurrent induction of apoptosis signaling, independent of cFLIP_L_ inhibitory expression (Figure 3G,H; Figure S3H–L). Altogether, these results suggest that intracellular TAT-FADD has the potential to induce both extrinsic and intrinsic apoptotic pathways, and counteracts the expression of anti-apoptotic proteins in cancer cells.

### 2.4. TAT-FADD Efficiently Induces Apoptosis Compared with Conventional Apoptosis Inducers

**Figure 4:**
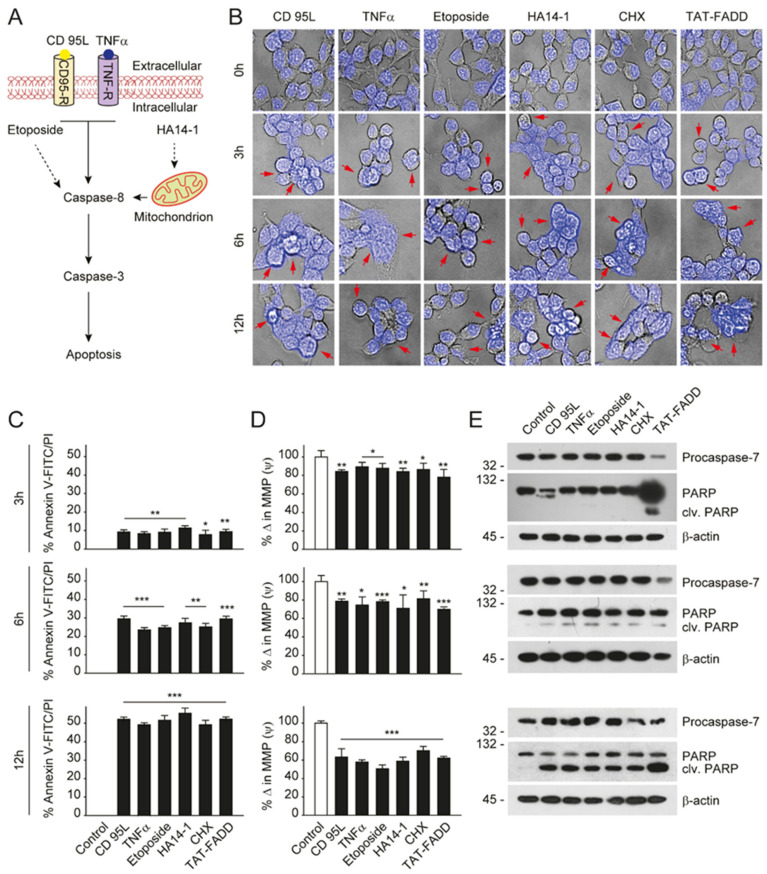
TAT-FADD’s pro-apoptotic effect is compared with conventional apoptosis inducers. (**A**) Schematic diagram representing the target site of proposed apoptosis inducers. (**B**–**E**) HCT 116 cells were treated with CD 95L (200 ng/mL), TNF-α (50 ng/mL), etoposide (50 µM), HA14-1 (5 µM), protein translational inhibitor cycloheximide (CHX, 5 µg/mL), and TAT-FADD (5 µM) alone for the mentioned time points, (**B**) The bright field images of cells counterstained with DAPI, post treatments, representative of 150 cells from 3 independent fields, scale bar 5 µm, (**C**) % apoptotic death by a Tali™ image-based cytometer, (**D**) % change in MMP and (**E**) expression of Procaspase-7 and cleavage of PARP by Western blot analysis, molecular weight marker left to each blot. In (**C**,**D**), significance compared between non-treated (0 h, white bars) and treated cells (black bars). h, hours; clv, cleaved; CD95-R, CD95 receptor; TNF-R, TNF receptor; MMP, mitochondrial membrane potential. Mean  ±  SD; * *p*  ≤  0.05, ** *p*  ≤  0.01, and *** *p*  ≤  0.001.

Given that TAT-FADD has the potential to induce both the caspase-8 and mitochondrial signaling of apoptosis, we next compared the apoptotic competency of TAT-FADD with conventional inducers of the death receptor and mitochondrial apoptosis. To this aim, we selected the death receptor ligands CD 95L and TNF-α; pro-apoptotic molecules, such as etoposide and HA14–1; and the protein translational inhibitor cycloheximide (CHX) (Figure 4A). The HCT 116 cells were treated with the mentioned molecules and TAT-FADD individually for 3–12 h. We found that within 3 h of treatment, TAT-FADD showed more disintegrated cellular morphology (shown with arrows) compared to the ligands or/and drugs, and remained at the maximum post 12 h of treatment (Figure 4B). Next, we measured cell viability and apoptotic death. We found that TAT-FADD death-inducing and pro-apoptotic activity was comparable with the death ligand CD 95L and pro-apoptotic molecule HA14-1, and post 12 h of incubation, TAT-FADD demonstrated maximum effect (Figure 4C; Figure S4A,B). We measured the mitochondrial membrane potential (MMP) and found a significant alteration in MMP of TAT-FADD-treated cells similar to CD 95L and HA14-1, post 3–12 h of treatment (Figure 4D). Next, we monitored the activation of PARP and caspase-7 as a confirmation of apoptotic instigation. Interestingly, at 3 h, we found processing and activation of PARP and caspases-7 only in TAT-FADD-treated cells as compared to remaining molecules. Moreover, at 6 and 12 h of incubation, the activation of PAPR and caspases-7 was comparable in all molecules and the same as TAT-FADD (Figure 4E). Moreover, we found that TAT-FADD synergistically enhances the pro-apoptotic effect when treated in combination with the death ligands CD95L or TNF-α in HCT116 cells (Figure S5A–D). Together, these results suggest that TAT-FADD rapidly induces apoptosis signaling and shows a similar pro-apoptotic response as observed with conventional death-inducing ligands and molecules.

### 2.5. TAT-FADD Mitigates NF-κB Activation in Cancer Cells

**Figure 5:**
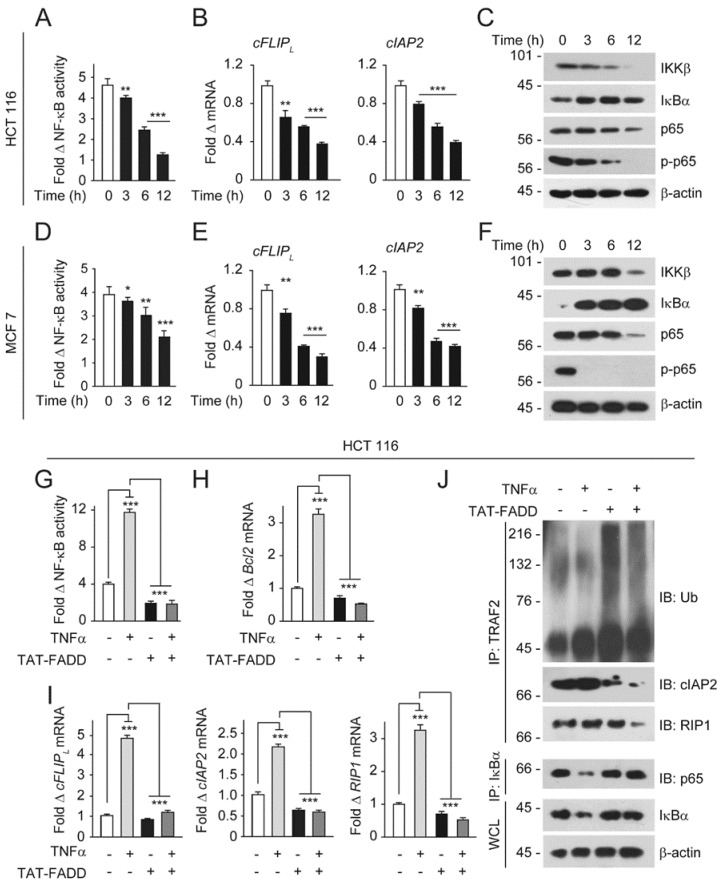
TAT-FADD suppresses constitutive and TNF-α-primed NF-κB activation in cancer cells. (**A**–**C**) HCT 116 cells were treated with 5 µM of TAT-FADD for 3–12 h and (**A**) NF-κB luciferase activity, (**B**) mRNA expression of *cFLIP_L_* and *cIAP2*, and (**C**) expression of NF-κB signaling protein, molecular weight marker left to each blot. (**D**–**F**) MCF-7 cells were treated with 5 µM of TAT-FADD for 3–12 h and (**D**) NF-κB luciferase activity, (**E**) mRNA expression of *cFLIP_L_* and *cIAP2*, and (**F**) expression of NF-κB signaling protein, molecular weight marker left to each blot. Control represents untreated cells. (**G**–**J**) HCT 116 cells were primed with TNF-α (10 ng/mL) for 12 h followed by treatment of 5 µM TAT-FADD for 6 h and (**G**) NF-κB luciferase activity, (**H**,**I**) mRNA expression of (**H**) *Bcl2* and (**I**) *cFLIP_L_*, *cIAP2* and *RIP1*, (**J**) TRAF2 was immunoprecipitated (IP) followed by binding analysis of ubiquitin, cIAP2, and RIP1 protein by Western blot, molecular weight marker left to each blot. Control represents untreated cells. In (**A**,**B**,**D**,**E**), significance compared between non-treated (represents as 0 h) and TAT-FADD-treated cells; in (**G**–**I**), significance is compared between non-treated (white bars) and TNF-α-primed cells (gray bars); and between TNF-α-primed cells and TAT-FADD-treated cells (black and dark gray bars respectively). h, hours; WCL, whole cell lysate. Mean  ±  SD; * *p*  ≤  0.05, ** *p*  ≤  0.01, and *** *p*  ≤  0.001.

Several studies investigated constitutive NF-κB activation in cancer cells [22,24,43]. The canonical activation of NF-κB is triggered upon ligation of TNF-α to its cognate receptor TNF receptor 1 (TNF-R1), leading to subsequent recruitment of TNF-R1-associated death domain (TRADD), FADD, TNF receptor-associated factor 2 (TRAF2), receptor-interacting protein kinase (RIP1), and the cellular inhibitor of apoptotic proteins (cIAPs) [44,45]. Moreover, downstream of the TRAF2-cIAPs-RIP1 complex, the IKKβ subunit of the IKK complex targets IκBα for polyubiquitination to facilitate release and nuclear translocation of NF-κB subunit p65 for subsequent transcriptional activations of pro-tumorigenic genes [46,47,48]. We and others have earlier reported that FADD regulates the activation of NF-κB signaling and downstream pathways [18,30]. Here, we asked whether direct delivery of TAT-FADD regulates core NF-κB signaling. We found a baseline activation of NF-κB signaling and expression of anti-apoptotic genes *cFLIP_L_* and *cIAP2* in cancer cells (Figure 5A–F). Next, we treated the cells with TAT-FADD and found that TAT-FADD significantly reduces NF-κB constitutive activation and expression of the anti-aopototic genes *cFLIP_L_*, *cIAP2*, *Bcl2*, and *RIP1* (Figure 5A,B,D,E; Figure S6A,B). Moreover, our results demonstrated that TAT-FADD regulates the IKKβ expression to up regulate IκBα expression, which in turn regulates p65 and phospho p65 levels (Figure 5C,F). Importantly, treatment of TAT-FADD significantly reduces the NF-κB activation and downstream expression of *Bcl2*, *cFLIP_L_*_,_ *cIAP2*, and *RIP1*, independent of TNF-α priming to these cells (Figure 5G–I). It is well established that anti-apoptotic cFLIP_L_ activates NF-κB signaling [49,50,51]. In an alternate approach, we examined the effect of TAT-FADD on reciprocal regulation of cFLIP_L_ to suppress NF-κB activation. We found that, in HCT116 cells, transient expression of cFLIP_L_ induces NF-κB activity; however, TAT-FADD potentially regulates cFLIP_L_ and induces NF-κB activation (Figure S6C). We found that TAT-FADD induces the ubiquitination and degradation of TARF2 complex, with a concomitant reduction in the binding of cIAP_2_ and RIP1 to TRAF2, independent of TNF-α stimulation (Figure 5J). Moreover, TAT-FADD treatment enhances the sequestering of IκBα and p65 to prevent nuclear translocation, independent of TNF-α priming to HCT 116 cells (Figure 5J). Altogether, these results suggest that TAT-FADD has the potential to target constitutive NF-κB signaling and abrogates TNF-α-mediated pro-survival NF-κB signaling in cancer cells.

### 2.6. TAT-FADD Restricts Inflammasome Components and Secretion of Proinflammatory Cytokine IL-1β

**Figure 6:**
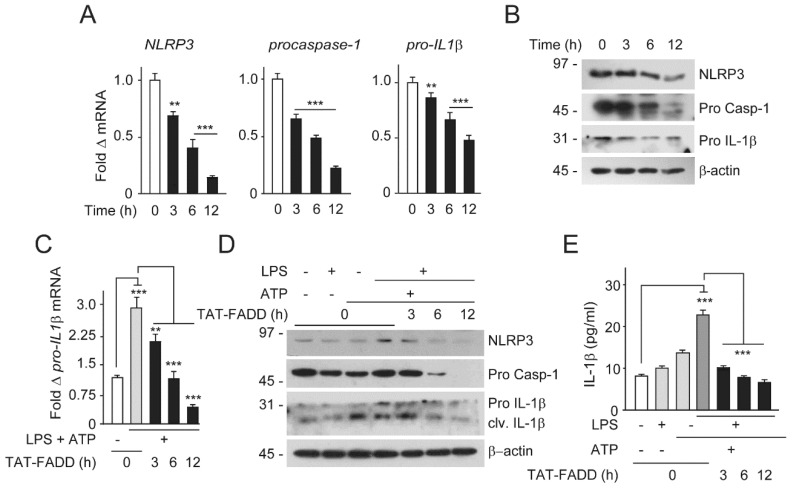
TAT-FADD suppresses proinflammatory activation of IL-1β. (**A**,**B**) HCT116 cells were treated with 5 µM of TAT-FADD for 3–12 h, (**A**) mRNA expression of *NLRP3*, *procaspase-1*, and *pro*-*IL-1β*, and (**B**) endogenous expression of NLRP3, procaspase-1 (pro Casp-1), and pro IL-1β by Western blot, molecular weight marker left to each blot. The 0 h represents untreated cells. (**C**–**E**) HCT 116 cells were primed with LPS (100 ng/mL, 12 h) and ATP (5 mM, 2 h) followed by treatment with 5 µM of TAT-FADD for 3–12 h and (**C**) mRNA expression of *pro-IL-1β*, (**D**) expression levels of endogenous NLRP3, procaspase-1, and pro IL-1β by Western blot, molecular weight marker left to each blot, and (**E**) levels of matured IL-1β was measured by ELISA assay from the culture supernatant. In (**C**,**E**) (white bar) and in (**D**) (lane 1), it represents un-primed and non-treated cells for 12 h taken as controls. In (**A**), significance is compared between non-treated (represents as 0 h) and TAT-FADD-treated cells; in (**C**), significance compared between non-treated (white bars) and LPS with ATP-primed cells (gray bars); and between LPS with ATP primed cells treated with TAT-FADD treated cells (black & dark gray bars respectively). h, hours. Mean  ±  SD; ** *p*  ≤  0.01 and *** *p*  ≤  0.001.

The NF-κB-activated transcriptional priming of *pro-IL1β* was further processed through the NLRP3 inflammasome complex to secret mature IL1β [28,29]. Given that TAT-FADD regulates NF-κB constitutive activation and downstream signaling, we asked whether TAT-FADD has the potential for regulation of NF-κB-induced priming of *pro-IL1β* and NLRP3 inflammasome signaling. We first examined the effect of TAT-FADD on the endogenous expression of NLRP3, procaspases-1, and pro-IL-1β. TAT-FADD treatment of HCT116 cells significantly suppressed the endogenous mRNA and protein expression of NLRP3, procaspase-1, and pro IL-1β with the progression of time (Figure 6A,B). Next, we primed HCT 116 cells with LPS followed by ATP treatment for the canonical activation of the NLRP3 inflammasome signaling, followed by TAT-FADD treatment. We found that TAT-FADD robustly acts on the expressions of *NLRP3*, *procaspse*-*1*, and *pro*-*IL1β*, independent of the NLRP3 inflammasome priming (Figure 6C; Figure S7). Moreover, we found that TAT-FADD treatment restricted the processing and maturation of IL-1β in HCT 16 cells, independent of the NLRP3 inflammasome priming (Figure 6D,E). Altogether, these results suggest that TAT-FADD robustly suppress the activation of NF-κB-induced proinflammatory priming and NLRP3 inflammasome-mediated processing of IL1β.

## 3. Discussion

The adaptor protein FADD provides scaffolding for the recruitment of various signaling proteins involves in distinct cellular pathways, including apoptosis and inflammation [1,52]. Mechanistically, FADD bridges the activated death receptor and the initiator caspases to augment cell death signaling [3,6]. However, in the majority of cancer cells, the cytosolic expression of FADD is typically suppressed [9,15,53,54,55]. This impediment is likely because of post-translational modifications (PTMs) and nuclear localization of FADD [9,10,56]. Apart from that, cancer cells have constitutive activation of pro-survival NF-κB signaling, which in turn activates anti-apoptotic proteins to impede FADD-mediated apoptosis signaling [26]. We and others have previously shown that FADD has the potential to reactivate apoptosis, if sufficiently expressed in cancer cells [18,34]. These studies highlighted that FADD could be a promising therapeutic molecule in cancer treatment.

Recent advancement of direct protein delivery to cancer cells demonstrated an attractive therapeutic strategy to develop protein-based drugs [36,37,38,57]. In this study, we chemically conjugated FADD protein with TAT peptide for delivery into cancer cells and investigated its effectiveness in instigating apoptosis signaling and regulation of NF-κB activation in cancer cells. Given the importance of FADD to resurge apoptosis signaling in cancer cells, earlier studies developed strategies, such as adenoviral [32,35] and attenuated bacterial-based vectors [34], for optimal intracellular expression of FADD, and observed induction of apoptosis. However, vector-based gene delivery systems are under the control of host-derived factors and possess limited control over expression [58]. In this study, we strategically stabilized purified FADD with chemical cross-linkers, and delivered an optimal concentration to cells in conjugation with TAT peptide for controlled release inside the cells. Similarly, previous studies demonstrated TAT encoding vectors for the exogenous delivery of proteins [59,60]. The strategists of TAT-mediated direct delivery of proteins in cancer cells have the advantage of independence from host transcriptional control and targeted delivery into cells. In line with the following observations, we found that TAT-conjugated FADD retained structural and functional competency as observed with the in vitro interaction assay and cytotoxic assay. The efficacy of protein delivery into cancer cells depends on the internalization mechanism and escape from intracellular degradation. In order to protect the endosomal degradation of conjugated cargoes, TAT peptide prefers caveolin-mediated endocytosis [41,42]. We found that TAT-FADD internalization was mediated through caveolar endosomes, as pharmacological blocking of caveolin restricted TAT-FADD entry to HCT116 cells. Additionally, in our experiments, we observed cytosolic retention of TAT-FADD during different time incubations. This is in coherence with previous studies, which have shown that induced expression of FADD was retained in the cytosol for apoptotic instigation [18,34].

Upon internalization, TAT-FADD interacts with pro-apoptotic caspase-8 and anti-apoptotic cFLIP_L_ protein to formulate DISC assembly in cancer cells, independent of death receptor activation to instigate downstream signaling [61]. Importantly, with the progression of time, a reduced interaction between cFLIP_L_ and TAT-FADD was observed in DISC assembly. This is similar to our previous report showing that FADD induces the degradation of cFLIP_L_ and recruits caspase-8 at DISC to instigate apoptosis signaling [18]. TAT-mediated delivery of pro-apoptotic proteins in combination with death-inducing compounds are more efficient in the sensitization of cancer cells [60]. In this study, we demonstrated that TAT-FADD significantly suppresses the viability of various origins of cancer and transformed cells, independent of pro-apoptotic stimuli. It is possible that each apoptosis-inducing protein has a different expression regulation and concentration-dependent effect in cancer cells [60,62]. Post DR stimulation, the immediate process is FADD-mediated constitution of DISC assembly for the downstream activation of apoptosis signaling [3,4], as we observed TAT-FADD readily formulated DISC assembly, independent of DR stimulation, and this was sufficient to induce apoptosis signaling in cancer cells. Our study provides a comparative analysis between TAT-FADD and conventional apoptosis inducers, as these inducers have distinct target and signaling mechanisms. TAT-FADD represents a pleiotropic molecule in the execution of both caspase-8 and mitochondrial-dependent apoptosis signaling.

Activated NF-κB signaling in cancer cells confines the efficacy of apoptosis inducers, leading to uncontrolled cell proliferation [22,23,63]. The expression of FADD in cancer cells is relatively low [13,15,54,55], and some studies have contributed that at low levels, FADD may contribute to NF-κB activation [64,65], while, seemingly in contrast to these studies, we and others reported that induced expression of FADD regulates NF-κB activation [18,30]. In this study, we found that TAT-FADD suppresses constitutive NF-κB activation in a time-dependent manner, and most importantly, TAT-FADD treatment targets the expression of NF-κB core signaling molecules to restrict their activation. Earlier studies demonstrated that the NF-κB downstream transcriptional gene *cFLIP_L_* has the potential to be activated upstream of NF-κB signaling in cancer cells [49,50,51]. In contrast, we showed that TAT-FADD significantly regulates *cFLIP_L_* and induces NF-κB activation. It is possible that TAT-FADD pro-apoptotic competency prevails over anti-apoptotic NF-κB signaling through regulation of the core molecules TRAF2, cIAP2, and RIP1. This is consistent with the finding that even TNF-α (NF-κB inducer)-primed colon carcinoma HCT116 cells failed to protect constitutive NF-κB activation, in response to TAT-FADD treatment. Based on these observations, we proposed that sufficient availability of intracellular FADD expression could potentially target NF-κB core signaling with a concurrent reduction of anti-apoptotic expression in cancer cells.

Consistent with anti-apoptotic activation, NF-κB-dependent transcriptional priming of proinflammatory genes further impedes apoptosis signaling in cancer cells [22,26,66]. Although, the exact mechanism of NF-κB-driven production of IL-1β in the regulation of apoptosis signaling is not clearly defined. An earlier study showed that genetic deletion of *FADD* leads to impaired IL1β processing and secretion in mouse myeloid cells [67]; however, in contrast to this study, another mouse myeloid cell study demonstrated that expression of FADD is critical for TLR4-induced transcriptional priming of the NLRP3 inflammasome and secretion of IL1β [68]. In this study, we found that treatment of HCT116 cells with TAT-FADD targeted the endogenous expression of the NLRP3 inflammasome complex molecules NLRP3 and caspase-1. These data suggest that in addition to targeting the NLRP3 inflammasome complex, TAT-FADD also reduces the proinflammatory priming and maturation of IL1β in HCT116 cells. Our study has some limitations. We did not examine the potential of TAT-FADD in in vivo tumor models; nevertheless, the effective outcomes of TAT-FADD, which we investigated in different origins of cancer and transformed cell lines, may provide a framework to design future in vivo studies. Based on our observations of TAT-FADD-mediated regulation of NF-κB signaling, it is possible that TAT-FADD contributes to the impediment of NF-κB-driven proinflammatory signaling. Further studies dissecting TAT-FADD-mediated regulation of inflammatory cell death would be interesting to investigate with an in vivo model system.

## 4. Materials and Methods

### 4.1. Materials

Molecular biology-grade reagents were purchased commercially. Poly-L lysine, protease inhibitor cocktail, H_2_DCFDA, DAPI, Flouramaount, SMCC (Succinimidyl- trans-4-(N-maleimidylmethyl)cyclohexane-1-carboxylate), HIV-TAT1, Iodocetamide, DMF, Cyclodextrin, Cycloheximide (CHX), Etoposide, Methyl β-cyclodextrin (MβCD), anti-His (SAB4301134), trypan blue, and a BCA protein estimation kit were purchased from Sigma-Aldrich (St. Louis, MO, USA). Ni^+2^-NTA beads, LipofectamineLTX plus transfection reagent Prestoblue viability assay kit, IL-1 beta Human ELISA Kit (BMS224HS), and Alexa fluors were purchased from Invitrogen (Life Technologies, Carlsbad, California, USA). A dual glow luciferase assay kit was purchased from Promega (Madison, WI, USA). The death receptor ligands CD 95L and TNF-α were obtained from ProSpec (Rehovot, Israel). HA14-1 was obtained from Maybridge (Cornwall, UK). The details of the antibodies used in this study are provided in Supplementary Material Table S1. All other chemicals used were of analytical grade and purchased from Merck (Darmstadt, Germany).

### 4.2. Cell Lines and In Vitro Cell Culture

The cell lines HCT 116, HEK 293, and RAW 264.7 were purchased from ATCC (Manassas, VA, USA) and MCF-7, HepG2, and HeLa cells were purchased from National Center for Cell Sciences, (Pune, India). HEK 293T, MCF-7, and HeLa cells were grown in Dulbecco’s Modified Eagle’s Medium (DMEM) and HCT 116, HT-29, and RAW 264.7 cells were grown in RPMI-1640 culture medium. All and culture media were supplemented with 10% fetal bovine serum, L-glutamine (2 mM), and antibiotic cocktail of penicillin (5 mg/mL), streptomycin (5 mg/mL), and neomycin (10 mg/mL) (GIBCO, Thermo Fisher Scientific, Waltham, MA, USA). The cells were cultured in a humidified CO_2_ incubator at 37 °C supplied with 95% O_2_ and 5% CO_2_. Exponentially growing cultured cells were used for experiments.

### 4.3. Cloning and Purification of Human FADD (hFADD)

Total RNA was isolated from HEK 293 cells with Trizol reagent according to the manufacturer’s protocol (Invitrogen, Carlsbad, California, USA). Then, 1 μg of total RNA was reversely transcribed to cDNA using the iScript cDNA Synthesis Kit (Bio-Rad Laboratories Inc., Hercules, CA, USA). The PCR amplicons of hFADD were subcloned into pET28a (+) (EMD Millipore, Burlington, MA, USA) and confirmed clones were transformed into *E. coli* BL21 (*DE3*)-pLys cells followed by induction with 0.5 mM IPTG for 2 h at 25 °C. The bacterial pellets were resuspended in the lysis buffer (50 mM sodium phosphate buffer, pH 8.0, 150 mM NaCl, 1 mM PMSF, and 0.1% Triton-X 100) followed by ultrasonication for 10 min on ice with a 2-min interval. The insoluble fraction was dissolved in protein refolding buffer (50 mM sodium phosphate buffer, pH 8.0, 150 mM NaCl, 6 M guanidium HCl, and 10 mM β-mercaptoethanol) and incubated overnight at 4 °C with gentle stirring. The refolded fractions were passed through a pre-equilibrated Ni^2+^ affinity column and the 6X His tag human FADD protein was purified and analyzed by SDS-PAGE and mass spectroscopy analysis. The purified His-hFADD was done with the BCA protein estimation kit (Sigma Aldrich) to determine the protein concentration.

### 4.4. Mass Spectrometry

The purified hFADD was characterized by MALDI-TOF analysis. In brief, a fraction containing purified hFADD protein from the SDS-PAGE gel was excised and incubated with 10 mM dithiotreitol (DTT) in 100 mM NH_4_HCO_3_ for 1 h at 56 °C. The reaction mixture was completely dried in a vacuum centrifuge and finally dissolved in the digestion buffer containing 50 mM NH_4_HCO_3_, 5 mM CaCl_2_, and 12.5 ng/µL of trypsin in an ice-cold bath. The (R-cyano-4-hydroxy-trans-cinnamic acid) and nitrocellulose matrix material were dissolved in acetone/2-propanol (1:1 *v*/*v*). Aliquots of 0.5 µL of analyte solution were deposited onto these matrix surfaces. A control sample included a fraction of the same SDS gel processed simultaneously. All mass spectra were obtained on a modified Bruker REFLEX mass spectrometer (Bruker Franzen Analytik, Bremen, Germany).

### 4.5. Chemical Conjugation of hFADD with TAT Peptide (TAT-FADD)

The purified FADD protein was conjugated with TAT peptide as per [69] with minor modifications. In brief, FADD protein at (25 mg/mL) was treated with iodoacetamide (final concentration, 0.37 mg/mL) for 1 h at 23 °C. Post incubation, the reaction mixture was incubated with linker SMCC (1.2 mg/mL, the final concentration 60 µg/mL) and incubated at room temperature for 30 min in the dark. The TAT peptide (200 µg/mL) was added the above reaction mixture and incubated overnight at 4 °C. The cross-linked conjugate of TAT-FADD was purified through the Ni^2+^ affinity column and the purity of the compound was confirmed by SDS-PAGE analysis.

### 4.6. Fourier-Transform Infrared Spectroscopy (FT-IR) Analysis

The chemical conjugation of human FADD with TAT peptide (TAT-FADD) was confirmed by FT-IR analysis. In brief, the spectra of iodoacetamide (ID), SMCC (MA), TAT (TT), purified FADD (FD), and TAT-FADD conjugate (TT-FD) were recorded in the wavelength range of 4000–700 cm^−1^ at a 4 cm^−1^ resolution and an interferogram of 64 scans was co-added to each sample. Spectral data were displayed in terms of transmission and viewed using Win-IR Pro Software (Agilent Technologies Inc., Santa Clara, CA, USA).

### 4.7. In Vitro Protein Interaction Assay

To characterize the binding of purified and/or TAT-conjugated FADD to partner proteins, recombinant His-tagged FADD (1 μg) was initially immunoprecipitated with anti-His (3 μg) and Sepharose A/G beads (Thermofisher Scientific, Waltham, MA, USA) in NP-40 buffer (20 mM Tris-HCl, pH 7.4, containing 150 mM NaCl, 0.2% (*vol*/*vol*) NP-40, 10% (*wt*/*vol*) glycerol, and complete protease-inhibitor ‘cocktail’). The protein-beads complexes were washed with binding buffer, followed by incubation with total cell lysates from HCT 116 (1 μg) overnight at 4 °C. The protein complex was eluted in SDS-PAGE sample buffer (62.5 mM Tris-HCl, pH 6.8, 10% (*wt*/*vol*) glycerol, 2% (*wt*/*vol*) SDS, 0.7 M β-mercaptoethanol, and 0.001% (*wt*/*vol*) bromophenol blue) and analyzed by Western blot.

### 4.8. Intracellular Examination of TAT-FADD

To examine the intracellular delivery of TAT-FADD, the HCT 116 and MCF7 cells at a density of 1 × 10^5^ cells/well were seeded on a cover slip kept in a 6-well plate and incubated for 16 h. The cells were transfected with 250 ng of GFP-Caveolin1 for 24 h. The GFP-Caveolin1-expressing cells were treated with 5 µM TAT-FADD as mentioned in the figure legends. Further cells were fixed with 4% paraformaldehyde and permeabilized with 0.5% Triton X-100. Post washes, cells were blocked with 1% BSA and incubated with primary antibody (anti-His as the FADD protein carries His tag) at a dilution of 1:50 overnight at 4 °C. Cover-slips were washed three times for 5 min each followed by incubation with secondary Alexa fluor 647 (1:100) for 1 h at room temperature in the dark. Cells were washed and mounted with Fluoromount mounting media. The cells were analyzed under a laser scanning confocal microscope (Leica SP5, Wetzlar, Germany). All the images were further analyzed and processed with Leica SP5 II software (Leica TCS SP5 II, Wetzlar, Germany). The percent co-localization was quantified with the aid of Image J software (NIH, Bethesda, MD, USA). In some experiments, the bright field images were captured through an inverted microscope (DP, 71, Olympus, Shinjuku-ku, Tokyo, Japan) and images were analyzed by image analysis software (Image-Pro MC 6.1, Bethesda, MD, USA).

### 4.9. Plasmid Constructs and Transfection

Plasmid expression vectors encoded pNFκ B-Luc: pGL3b-kB4/pRL-TK: Renilla Luciferase (provided by Dr. Susan Nozell, University of Alabama at Birmingham, Birmingham, AL, USA) and pEGFP-caveolin-1 (provided by Prof. David Le Couteur, University of Sydney, NSW, Australia). The cDNA encoding for cFLIP_L_ (original vector pLXSN-cFLIP_L_, provided by Prof. Sabine Adam-Klages, Universitätsklinium Schleswig-Holstein, Kiel, Germany) was further subcloned into mammalian expression vector pcDNA3 (Invitrogen). All the constructs were transfected into mentioned cells using lipofectamine LTX plus transfection reagent (Life Technologies), according to the manufacturer’s instructions.

### 4.10. Cell Viability and Toxicity and Apoptotic Cell Death Analysis

In brief, cells were seeded at a density of 2 × 10^4^ cells in a 96-well plate and incubated for 24 h. Cells were subjected to treatments as mentioned in the figure legends. The percent cell viability was examined by a Prestoblue cell viability assay kit (Life Technology) according to the manufacturer’s instructions. The release of LDH was analyzed by an LDH cytotoxicity detection kit (MK401, Takara-Clontech, Mountain View, CA, USA) as per the manufacturer’s instructions. The result represents the percentage release of LDH. In some experiments, cells were stained with an Annexin-V FITC/PI staining kit (BioVision, Mountain View, CA, USA) and percent apoptotic death was analyzed by a Tali™ image-based cytometer (Life Technologies) and qualitative analysis was carried out under a fluorescent microscope (DP71, Olympus, Japan).

### 4.11. Flow Cytometry for Apoptotic Death Analysis

In brief, HCT 116 cells (2  ×  10^6^ cells) were subjected to treatments as mentioned in the figure legends followed by incubation in 1X Annexin binding buffer and Annexin-V-FITC and propidiumiodide solution as per the manufacturer’s instructions (BD Biosciences, San Jose, CA, USA). Cells were acquired and analyzed by BD FACSDiva software (BD Biosciences). The results are represented in contour plots with quadrant gates showing early apoptosis in quadrant 4 (Q4) and late apoptosis in quadrant 2 (Q2).

### 4.12. Isolation of RNA and Real-Time-qPCR

Total RNA was extracted using Trizol reagent (Invitrogen) and reverse transcribed into cDNA using the iScript cDNA Synthesis Kit as per the manufacturer’s instructions (Bio-Rad Laboratories). The cDNA was then amplified and analyzed by RT-qPCR as previously described [19]. The primer sequences of the respective genes are provided in Supplementary Material Table S2. Each assay was normalized by using the difference in critical thresholds (C_T_) between target genes and 18SrRNA. The expression of mRNA of respective genes was compared with the control using the values of 2^−ΔΔCT^.

### 4.13. Subcellular Fractions

In brief, 1 × 10^6^ cells were seeded in a 60-mm dish and incubated for16 h. Cells were treated with 5 µM TAT-FADD for 3–12. Post treatment, cells were harvested and resuspended in hypotonic buffer (10 mM HEPES pH 7.5, 10 mM Kcl, 1.5 mM MgCl_2_, 0.1 mM DTT, 0.5% Triton X-100, 1 mM PMSF, and 1X PIC) for 5 min on ice and spun at 3000 rpm for 2 min at 4 °C. The supernatant was stored as “cytoplasmic fraction”, and the pellets were washed with wash buffer (10 mM HEPES pH 7.5, 10 mM KCl, 1.5 mM MgCl_2_, 0.1 mM DTT, 1 mM PMSF, and 1X PIC). Subsequently, pellets were lysed with lysis buffer (150 mM NaCl, 1% Triton X-100, 0.5% Sodium deoxycholate, 0.1% SDS, 50 mM Tris, pH 8.0, 1 mM PMSF, 1 mM NaV, and 1X PIC) for 25 min on ice and spun at 10,000 rpm for 15 min. The supernatant were stored as “nuclear fraction”. The protein concentrations of both the fractions was estimated by a BCA protein estimation kit (Sigma-Aldrich); 50 μg of protein sample were subjected to Western blot analysis.

### 4.14. Co-Immunoprecipitation Assay

In brief, 2 × 10^6^ cells were subjected to treatments as mentioned in the figure legends. The cells were lysed in NP-40lysis buffer (20 mM Tris-HCl, pH 7.4, 150 mM NaCl, 10% glycerol, 0.2% Nonidet P40, and 1X protease inhibitor mixture (Roche, Basel, Switzerland) and the protein concentration was estimated by the BCA protein estimation kit (Sigma, St. Louis, MO, USA). Next, 350 µg of protein were incubated with 2 µg of respective antibodies (as mentioned in the figure legends) with 20 µL of SepharoseA/G beads overnight at 4 °C with gentle shaking. The beads were washed with RIPA buffer (50 mM Tris-HCl (pH 7.4), 150 mM NaCl, 10% glycerol, 1% triton-X 100, 0.5% Na-deoxycholate, 0.1% SDS, and 1X protease inhibitor mixture) and the protein samples were fractionated on 8–12% SDS-PAGE followed by Western blot.

### 4.15. Western Blotting

In brief, cells were seeded at a density of 4 × 10^5^ cells in a 6-well plate and incubated for 24 h. Cells were subjected to treatments as mentioned in the figure legends and processed as described earlier (Ranjan et al., 2014) [70]. The membrane was blocked with 5% non-fat milk in Tris-buffered saline for 3 h at room temperature (for phospho protein, 5% BSA was used) followed by overnight incubation with primary antibody of anti-His (1:1000), anti-cFLIP_L_ (1:500), anti-p53 (1:500), anti-procaspase-8 (1:500), anti-procaspasse-7 (1:500), anti-procaspase-9 (1:1000), anti-Bcl2 (1:500), anti-PARP (1:1000), anti-cytochrome C (1:500), anti-TRAF2 (1:500), anti-p65 (1:1000), anti- phospho p65 (1:1000), anti-IκBα (1:1000), anti-ubiquitin (1:1000), anti-RIP1 (1:1000), anti-cIAP2 (1:250), anti-IKKβ (1:500) anti-procaspase-1 (1:1000), anti-pro IL-1β (1:250), anti-Nlrp3 (1:1000), and anti-β-actin (1:2000) at 4 °C. Post incubation, membranes were washed and probed with HRP-conjugated secondary antibodies (1: 10,000). The membranes were incubated with a Novex^®^ ECL HRP-linked chemiluminiscent substrate kit as per the manufacturer’s instructions (Invitrogen) and developed in Kodak X-Omat blue film (NEN Life Sciences, Inc., Boston, MA, USA).

### 4.16. NF-κB Luciferase Reporter Assay

In brief, 1 × 10^5^ cells were co-transfected with pGL3b-kB4, pRL-TK, and promoter-Renilla luciferase reporter plasmids using lipofectamine LTX plus transfection reagent (Invitrogen) according to the manufacturer’s instructions. Cells were subjected to treatments as mentioned in the figure legends and the NF-κB luciferase assay was performed with a Luciferase Assay Kit as per the manufacturer’s instructions (Promega). The results represent the fold change in NF-κB luciferase activity.

### 4.17. Measurement of Caspase 8 and Caspase 3 Activity

The fold induction of caspase-8 activity was determined by a Caspase-8/FLICE fluorometric assay kit (Biovision) as previously described [19]. Next, the fold induction caspase-3 activity was determined by an EnzChek Caspase-3 Assay Kit (Life Technologies) as previously described [70]. The result represents the fold activity of caspase-3 with respect to control cells.

### 4.18. Measurement of Mitochondrial Membrane Potential (ΔΨm)

Cells were subjected to treatments as mentioned in the figure legends and the change in mitochondrial membrane potential (ΔΨm) was determined by using JC-1 fluorescent dye (BioVision) as previously described [70]. The results represent the fold change in mitochondrial membrane potential.

### 4.19. Enzyme-Linked Immunosorbent Assays (ELISA)

Cells were subjected to treatments as mentioned in the figure legends. The concentrations of IL-1β secreted in culture media were determined by an ELISA development kit according to the manufacturer’s protocol.

### 4.20. Statistical Analysis

Statistical analysis was performed by one-way analysis of variance (ANOVA) followed by a student Newman Keulas test for multiple comparison and student t-test using SigmaStat statistical analysis software. Values were expressed as mean ±S.E.M. from three independent experiments. Differences were considered statistically significant at * *p* ≤ 0.05.

## 5. Conclusions

In conclusion, our study highlights the importance of FADD in the regulation of NF-κB-driven anti-apoptotic and proinflammatory signaling. We proposed an efficient strategy to deliver FADD protein in a controlled manner with the better targeting ability of cancer cells and this therapeutic strategy may be useful to overcome temporal and uncontrolled expression modulation as observed with the conventional gene delivery approach.

## Supplementary Materials

The following are available online at https://www.mdpi.com/1422-0067/21/18/6890/s1, Figure S1: Purification of human FADD protein and TAT-FADD conjugates; Figure S2: TAT-FADD significantly reduces the viability of cancer and transformed cells; Figure S3: TAT-FADD induced apoptosis signaling; Figure S4: TAT-FADD comparisions with conventional apoptosis inducers.; Figure S5: TAT-FADD synergistically enhances death ligands apoptotic competency; Figure S6: TAT-FADD suppresses NF-κB signaling downstream genes in cancer cells; Figure S7: TAT-FADD suppresses NLRP3 inflammasome primed activation; Table S1: List of antibodies used in the study; Table S2: List of real time PCR primers used in the study.

## References

[1] Mouasni S., Tourneur L. (2018). Fadd at the crossroads between cancer and inflammation. Trends Immunol..

[2] Werner M.H., Wu C., Walsh C.M. (2006). Emerging roles for the death adaptor fadd in death receptor avidity and cell cycle regulation. Cell Cycle.

[3] Scott F.L., Stec B., Pop C., Dobaczewska M.K., Lee J.J., Monosov E., Robinson H., Salvesen G.S., Schwarzenbacher R., Riedl S.J. (2009). The fas-fadd death domain complex structure unravels signalling by receptor clustering. Nature.

[4] Lavrik I., Krueger A., Schmitz I., Baumann S., Weyd H., Krammer P.H., Kirchhoff S. (2003). The active caspase-8 heterotetramer is formed at the cd95 disc. Cell Death Differ..

[5] Ho P.K., Hawkins C.J. (2005). Mammalian initiator apoptotic caspases. FEBS J..

[6] Julien O., Wells J.A. (2017). Caspases and their substrates. Cell Death Differ..

[7] Okano H., Shiraki K., Inoue H., Kawakita T., Yamanaka T., Deguchi M., Sugimoto K., Sakai T., Ohmori S., Fujikawa K. (2003). Cellular flice/caspase-8-inhibitory protein as a principal regulator of cell death and survival in human hepatocellular carcinoma. Lab. Investig..

[8] Ram D.R., Ilyukha V., Volkova T., Buzdin A., Tai A., Smirnova I., Poltorak A. (2016). Balance between short and long isoforms of cflip regulates fas-mediated apoptosis in vivo. Proc. Natl. Acad. Sci. USA.

[9] Alappat E.C., Feig C., Boyerinas B., Volkland J., Samuels M., Murmann A.E., Thorburn A., Kidd V.J., Slaughter C.A., Osborn S.L. (2005). Phosphorylation of fadd at serine 194 by ckialpha regulates its nonapoptotic activities. Mol. Cell.

[10] Gomez-Angelats M., Cidlowski J.A. (2003). Molecular evidence for the nuclear localization of fadd. Cell Death Differ..

[11] Lee E.W., Kim J.H., Ahn Y.H., Seo J., Ko A., Jeong M., Kim S.J., Ro J.Y., Park K.M., Lee H.W. (2012). Ubiquitination and degradation of the fadd adaptor protein regulate death receptor-mediated apoptosis and necroptosis. Nat. Commun..

[12] Chen G., Bhojani M.S., Heaford A.C., Chang D.C., Laxman B., Thomas D.G., Griffin L.B., Yu J., Coppola J.M., Giordano T.J. (2005). Phosphorylated fadd induces nf-kappab, perturbs cell cycle, and is associated with poor outcome in lung adenocarcinomas. Proc. Natl. Acad. Sci. USA.

[13] Marin-Rubio J.L., Vela-Martin L., Fernandez-Piqueras J., Villa-Morales M. (2019). Fadd in cancer: Mechanisms of altered expression and function, and clinical implications. Cancers.

[14] Tourneur L., Delluc S., Levy V., Valensi F., Radford-Weiss I., Legrand O., Vargaftig J., Boix C., Macintyre E.A., Varet B. (2004). Absence or low expression of fas-associated protein with death domain in acute myeloid leukemia cells predicts resistance to chemotherapy and poor outcome. Cancer Res..

[15] Tourneur L., Mistou S., Michiels F.M., Devauchelle V., Renia L., Feunteun J., Chiocchia G. (2003). Loss of fadd protein expression results in a biased fas-signaling pathway and correlates with the development of tumoral status in thyroid follicular cells. Oncogene.

[16] Soung Y.H., Lee J.W., Kim S.Y., Nam S.W., Park W.S., Kim S.H., Lee J.Y., Yoo N.J., Lee S.H. (2004). Mutation of fadd gene is rare in human colon and stomach cancers. APMIS.

[17] Korkolopoulou P., Saetta A.A., Levidou G., Gigelou F., Lazaris A., Thymara I., Scliri M., Bousboukea K., Michalopoulos N.V., Apostolikas N. (2007). C-flip expression in colorectal carcinomas: Association with fas/fasl expression and prognostic implications. Histopathology.

[18] Ranjan K., Pathak C. (2016). Fadd regulates nf-kappab activation and promotes ubiquitination of cflipl to induce apoptosis. Sci. Rep..

[19] Ranjan K., Surolia A., Pathak C. (2012). Apoptotic potential of fas-associated death domain on regulation of cell death regulatory protein cflip and death receptor mediated apoptosis in hek 293t cells. J. Cell Commun. Signal..

[20] Ranjan K., Pathak C. (2016). Expression of fadd and cflipl balances mitochondrial integrity and redox signaling to substantiate apoptotic cell death. Mol. Cell. Biochem..

[21] Li Q., Verma I.M. (2002). Nf-kappab regulation in the immune system. Nat. Rev. Immunol..

[22] Xia Y., Shen S., Verma I.M. (2014). Nf-kappab, an active player in human cancers. Cancer Immunol. Res..

[23] Eluard B., Thieblemont C., Baud V. (2020). Nf-kappab in the new era of cancer therapy. Trends Cancer.

[24] Nagel D., Vincendeau M., Eitelhuber A.C., Krappmann D. (2014). Mechanisms and consequences of constitutive nf-kappab activation in b-cell lymphoid malignancies. Oncogene.

[25] Sau A., Lau R., Cabrita M.A., Nolan E., Crooks P.A., Visvader J.E., Pratt M.A. (2016). Persistent activation of nf-kappab in brca1-deficient mammary progenitors drives aberrant proliferation and accumulation of DNA damage. Cell Stem Cell.

[26] Taniguchi K., Karin M. (2018). Nf-kappab, inflammation, immunity and cancer: Coming of age. Nat. Rev. Immunol..

[27] He Y., Hara H., Nunez G. (2016). Mechanism and regulation of nlrp3 inflammasome activation. Trends Biochem. Sci..

[28] Martinon F., Burns K., Tschopp J. (2002). The inflammasome: A molecular platform triggering activation of inflammatory caspases and processing of proil-beta. Mol. Cell.

[29] Schroder K., Tschopp J. (2010). The inflammasomes. Cell.

[30] Bannerman D.D., Tupper J.C., Kelly J.D., Winn R.K., Harlan J.M. (2002). The fas-associated death domain protein suppresses activation of nf-kappa b by lps and il-1 beta. J. Clin. Investig..

[31] Mouasni S., Gonzalez V., Schmitt A., Bennana E., Guillonneau F., Mistou S., Avouac J., Ea H.K., Devauchelle V., Gottenberg J.E. (2019). The classical nlrp3 inflammasome controls fadd unconventional secretion through microvesicle shedding. Cell Death Dis..

[32] Kondo S., Ishizaka Y., Okada T., Kondo Y., Hitomi M., Tanaka Y., Haqqi T., Barnett G.H., Barna B.P. (1998). Fadd gene therapy for malignant gliomas in vitro and in vivo. Hum. Gene Ther..

[33] Komata T., Koga S., Hirohata S., Takakura M., Germano I.M., Inoue M., Kyo S., Kondo S., Kondo Y. (2001). A novel treatment of human malignant gliomas in vitro and in vivo: Fadd gene transfer under the control of the human telomerase reverse transcriptase gene promoter. Int. J. Oncol..

[34] Yang Y.W., Zhang C.M., Huang X.J., Zhang X.X., Zhang L.K., Li J.H., Hua Z.C. (2016). Tumor-targeted delivery of a c-terminally truncated fadd (n-fadd) significantly suppresses the b16f10 melanoma via enhancing apoptosis. Sci. Rep..

[35] Kobayashi T., Okamoto K., Kobata T., Hasunuma T., Kato T., Hamada H., Nishioka K. (2000). Novel gene therapy for rheumatoid arthritis by fadd gene transfer: Induction of apoptosis of rheumatoid synoviocytes but not chondrocytes. Gene Ther..

[36] Lee Y.W., Luther D.C., Kretzmann J.A., Burden A., Jeon T., Zhai S., Rotello V.M. (2019). Protein delivery into the cell cytosol using non-viral nanocarriers. Theranostics.

[37] Liu X., Wu F., Ji Y., Yin L. (2019). Recent advances in anti-cancer protein/peptide delivery. Bioconjug. Chem..

[38] Zhang Y., Roise J.J., Lee K., Li J., Murthy N. (2018). Recent developments in intracellular protein delivery. Curr. Opin. Biotechnol..

[39] Jafari B., Pourseif M.M., Barar J., Rafi M.A., Omidi Y. (2019). Peptide-mediated drug delivery across the blood-brain barrier for targeting brain tumors. Expert Opin. Drug Deliv..

[40] Jones S.W., Christison R., Bundell K., Voyce C.J., Brockbank S.M., Newham P., Lindsay M.A. (2005). Characterisation of cell-penetrating peptide-mediated peptide delivery. Br. J. Pharm..

[41] Ferrari A., Pellegrini V., Arcangeli C., Fittipaldi A., Giacca M., Beltram F. (2003). Caveolae-mediated internalization of extracellular hiv-1 tat fusion proteins visualized in real time. Mol. Ther..

[42] Fittipaldi A., Ferrari A., Zoppe M., Arcangeli C., Pellegrini V., Beltram F., Giacca M. (2003). Cell membrane lipid rafts mediate caveolar endocytosis of hiv-1 tat fusion proteins. J. Biol. Chem..

[43] Staudt L.M. (2010). Oncogenic activation of nf-kappab. Cold Spring Harb. Perspect. Biol..

[44] Golks A., Brenner D., Fritsch C., Krammer P.H., Lavrik I.N. (2005). C-flipr, a new regulator of death receptor-induced apoptosis. J. Biol. Chem..

[45] Micheau O., Tschopp J. (2003). Induction of tnf receptor i-mediated apoptosis via two sequential signaling complexes. Cell.

[46] Chen Z.J. (2005). Ubiquitin signalling in the nf-kappab pathway. Nat. Cell Biol..

[47] Ea C.K., Deng L., Xia Z.P., Pineda G., Chen Z.J. (2006). Activation of ikk by tnfalpha requires site-specific ubiquitination of rip1 and polyubiquitin binding by nemo. Mol. Cell.

[48] Yamamoto Y., Gaynor R.B. (2004). Ikappab kinases: Key regulators of the nf-kappab pathway. Trends Biochem. Sci..

[49] Baratchian M., Davis C.A., Shimizu A., Escors D., Bagneris C., Barrett T., Collins M.K. (2016). Distinct activation mechanisms of nf-kappab regulator inhibitor of nf-kappab kinase (ikk) by isoforms of the cell death regulator cellular flice-like inhibitory protein (cflip). J. Biol. Chem..

[50] Kataoka T., Tschopp J. (2004). N-terminal fragment of c-flip(l) processed by caspase 8 specifically interacts with traf2 and induces activation of the nf-kappab signaling pathway. Mol. Cell. Biol..

[51] Golks A., Brenner D., Krammer P.H., Lavrik I.N. (2006). The c-flip-nh2 terminus (p22-flip) induces nf-kappab activation. J. Exp. Med..

[52] Tourneur L., Chiocchia G. (2010). Fadd: A regulator of life and death. Trends Immunol..

[53] Cimino Y., Costes A., Damotte D., Validire P., Mistou S., Cagnard N., Alifano M., Regnard J.F., Chiocchia G., Sautes-Fridman C. (2012). Fadd protein release mirrors the development and aggressiveness of human non-small cell lung cancer. Br. J. Cancer.

[54] Marin-Rubio J.L., de Arriba M.C., Cobos-Fernandez M.A., Gonzalez-Sanchez L., Ors I., Sastre I., Fernandez-Piqueras J., Villa-Morales M. (2016). Deregulated fadd expression and phosphorylation in t-cell lymphoblastic lymphoma. Oncotarget.

[55] Marin-Rubio J.L., Perez-Gomez E., Fernandez-Piqueras J., Villa-Morales M. (2019). S194-p-fadd as a marker of aggressiveness and poor prognosis in human t-cell lymphoblastic lymphoma. Carcinogenesis.

[56] Foger N., Bulfone-Paus S., Chan A.C., Lee K.H. (2009). Subcellular compartmentalization of fadd as a new level of regulation in death receptor signaling. FEBS J..

[57] Stewart M.P., Sharei A., Ding X., Sahay G., Langer R., Jensen K.F. (2016). In vitro and ex vivo strategies for intracellular delivery. Nature.

[58] Goverdhana S., Puntel M., Xiong W., Zirger J.M., Barcia C., Curtin J.F., Soffer E.B., Mondkar S., King G.D., Hu J. (2005). Regulatable gene expression systems for gene therapy applications: Progress and future challenges. Mol. Ther..

[59] Essafi M., Baudot A.D., Mouska X., Cassuto J.P., Ticchioni M., Deckert M. (2011). Cell-penetrating tat-foxo3 fusion proteins induce apoptotic cell death in leukemic cells. Mol. Cancer Ther..

[60] Orzechowska E.J., Kozlowska E., Czubaty A., Kozlowski P., Staron K., Trzcinska-Danielewicz J. (2014). Controlled delivery of bid protein fused with tat peptide sensitizes cancer cells to apoptosis. BMC Cancer.

[61] Siegel R.M., Frederiksen J.K., Zacharias D.A., Chan F.K., Johnson M., Lynch D., Tsien R.Y., Lenardo M.J. (2000). Fas preassociation required for apoptosis signaling and dominant inhibition by pathogenic mutations. Science.

[62] Orzechowska E.J., Girstun A., Staron K., Trzcinska-Danielewicz J. (2015). Synergy of bid with doxorubicin in the killing of cancer cells. Oncol. Rep..

[63] Karin M., Lin A. (2002). Nf-kappab at the crossroads of life and death. Nat. Immunol..

[64] Chaudhary P.M., Eby M.T., Jasmin A., Kumar A., Liu L., Hood L. (2000). Activation of the nf-kappab pathway by caspase 8 and its homologs. Oncogene.

[65] Hu W.H., Johnson H., Shu H.B. (2000). Activation of nf-kappab by fadd, casper, and caspase-8. J. Biol. Chem..

[66] Smale S.T. (2011). Hierarchies of nf-kappab target-gene regulation. Nat. Immunol..

[67] Bossaller L., Chiang P.I., Schmidt-Lauber C., Ganesan S., Kaiser W.J., Rathinam V.A., Mocarski E.S., Subramanian D., Green D.R., Silverman N. (2012). Cutting edge: Fas (cd95) mediates noncanonical il-1beta and il-18 maturation via caspase-8 in an rip3-independent manner. J. Immunol..

[68] Gurung P., Anand P.K., Malireddi R.K., Vande Walle L., Van Opdenbosch N., Dillon C.P., Weinlich R., Green D.R., Lamkanfi M., Kanneganti T.D. (2014). Fadd and caspase-8 mediate priming and activation of the canonical and noncanonical nlrp3 inflammasomes. J. Immunol..

[69] Fawell S., Seery J., Daikh Y., Moore C., Chen L.L., Pepinsky B., Barsoum J. (1994). Tat-mediated delivery of heterologous proteins into cells. Proc. Natl. Acad. Sci. USA.

[70] Ranjan K., Sharma A., Surolia A., Pathak C. (2014). Regulation of ha14-1 mediated oxidative stress, toxic response, and autophagy by curcumin to enhance apoptotic activity in human embryonic kidney cells. BioFactors.

