# Supplementary material for: Cell-Penetrable Peptide-Conjugated FADD Induces Apoptosis and Regulates Inflammatory Signaling in Cancer Cells
Source: Int J Mol Sci. 2020 Sep 19;21(18):6890. doi: 10.3390/ijms21186890 (PMC7555701; doi:10.3390/ijms21186890)
Supplement: Supplementary file 1 [file ijms-21-06890-s001.pdf]

International Journal of
Molecular Sciences

MDPI

Supplementary Figures

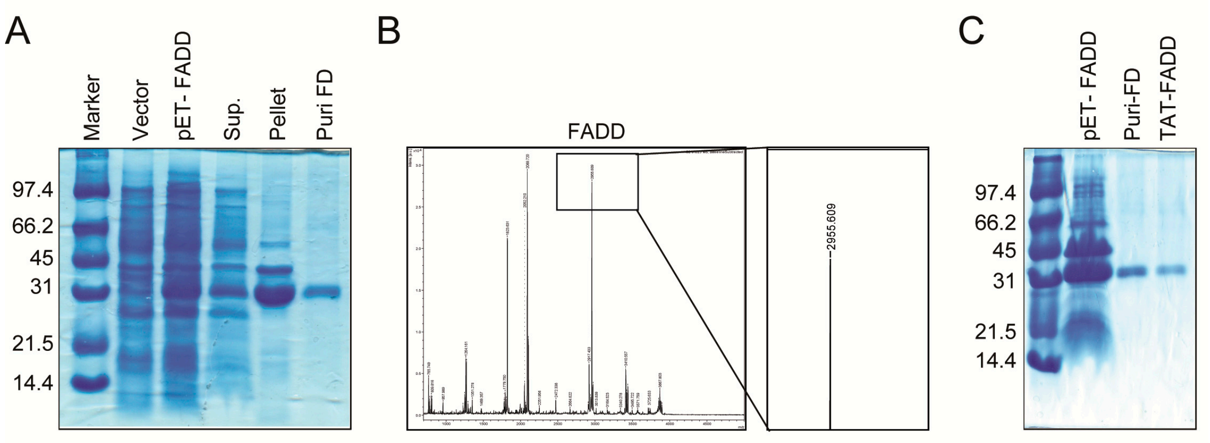

Figure S1. Purification of human FADD protein and TAT-FADD conjugates. (**A**) The SDS-PAGE gel of total lysate from vector (lane 2), IPTG induced pET-FADD (lane 3) transformed cells, cells from lane 3 subjected to sonication, loading of supernatant (sup.; lane 4) and pellet (lane 5), and purified FADD fraction (puri. FD, lane 5), molecular weight marker (lane 1). (**B**) Mass spectrometry analysis of purified FADD, the observed m/z of purified FADD protein at 2955.609 Da, enlarged in the inset box of representative spectrum. (**C**) The SDS-PAGE gel of IPTG induced lysate from pET-FADD transformed cells (lane 2), purified FADD (lane 3) and purified TAT-FADD (lane 4), molecular weight marker (lane 1).

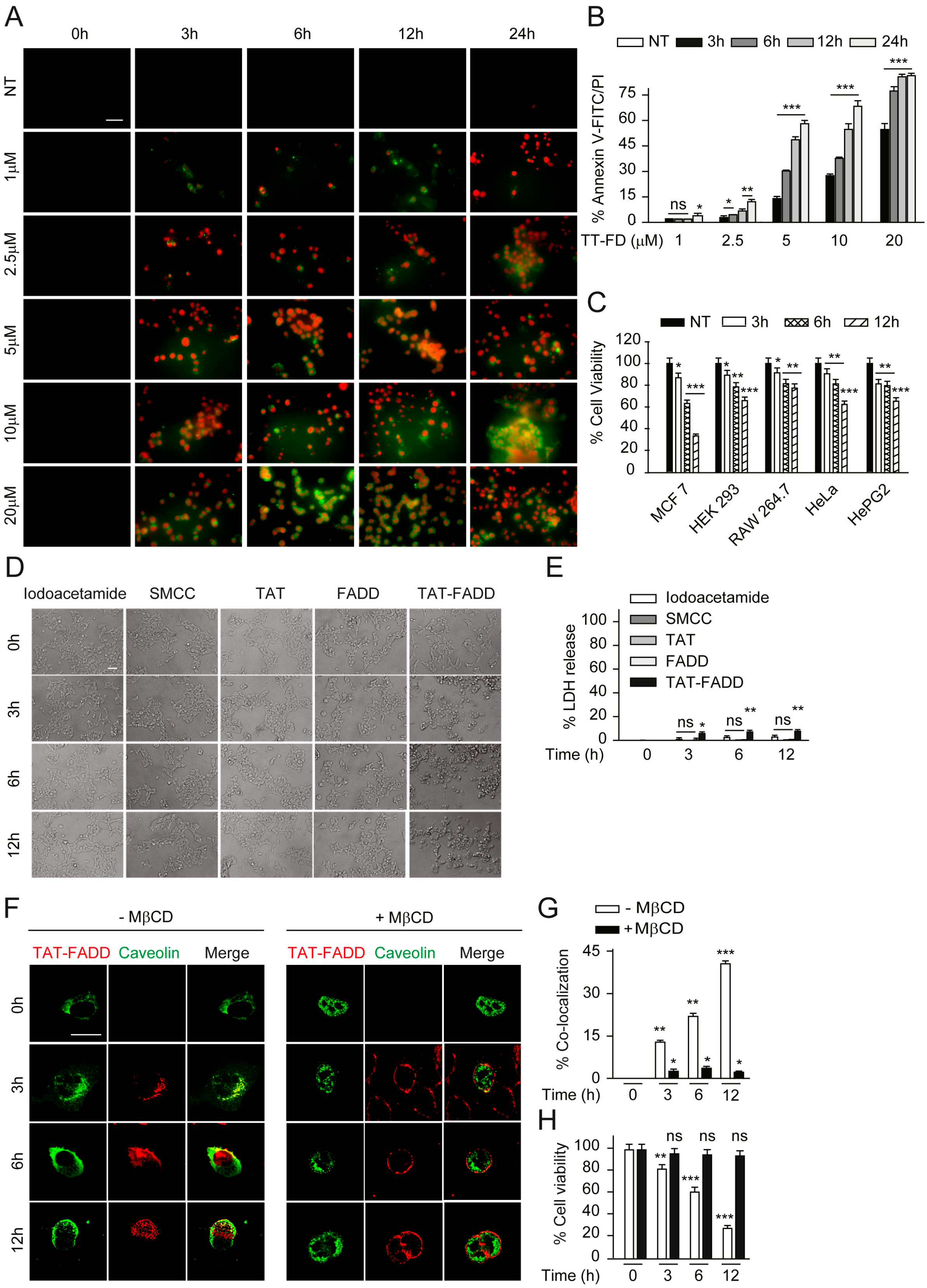

Figure S2. TAT-FADD significantly reduces the viability of cancer and transformed cells. (**A–B**) HCT 116 cells were treated with TAT-FADD (TT-FD) as mentioned concentrations for given time points and, (**A**) Cells were stained with Annexin-PI staining kit, and analyzed by fluorescent microscoe, representative of 150 cells from 3 different fields; scale bar 5 μm and (**B**) % apoptotic death by Tali™ image based cytometer, control represents non-treated (NT) cells at 0 h. (**C**) MCF-7,

HEK293, RAW 264.7, HeLa and HePG2 cells were treated with 5 μM of TAT-FADD for 3–12 h and analysis of cell viability. Control (black bar) represents non- treated (NT) cells. (**D**–**E**) HCT 116 cells were treated with 0.37 mg/mL iodoacetamide, 60 μg/mL SMCC, 200 μg/mL TAT peptide, 5 μM purified FADD protein and 5 μM TAT-FADD for 3–12 h followed by analysis of (**D**) bright field images of cells, post treatments, representative of 150 cells from 3 independent fields, scale bar 2 μm and (**E**) % LDH release in culture medium, post treatment. The 0h represents untreated cells taken as controls. (**F**–**H**) MCF-7 cells were transfected with GFP-Caveolin1 for 24 h followed by pre-incubation with MβCD (+MβCD; right panel) for 4 h, further cells were left untreated (0 h) or treated with 5 μM of TAT-FADD for 3–12 h, (**F**) the internalized TAT-FADD was immunostained with anti-His antibody (His tagged FADD) followed by counterstaining with DAPI and analyzed by confocal microscopy, representative of 25 cells from 3 different fields; scale bar 10 μm and (**G**) the % localization of TAT-FADD with GFP-Caveolin1 from 'C' was calculated with Image J software, (H) analysis of cell viability in the absence (-) and presence (+) of MβCD. In **B**, **C** & **E** significance compared between non-treated (NT and 0 h) and treated cells. In **G** & **H** significance compared between unprimed (-MβCD; white bars) and primed (+MβCD; black bars) cells treated with TAT-FADD, h, hours; ns, non-significant. Mean ± SD; $^{*}p \leq 0.05$, $^{**}p \leq 0.01$ and $^{***}p \leq 0.001$.

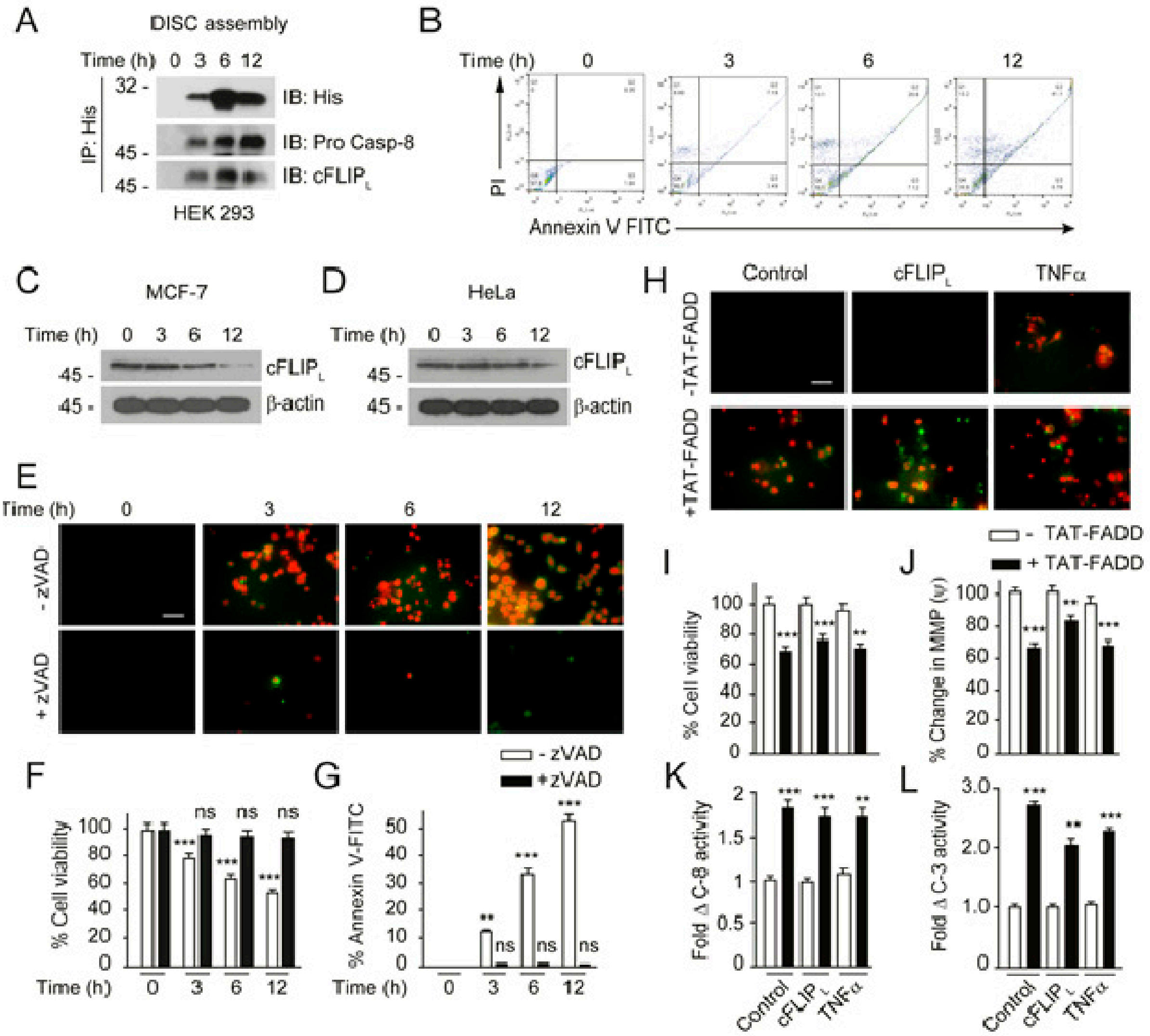

Figure S3. TAT-FADD induced apoptosis signaling. (**A**) HEK 293 cells were treated with 5 μM of TAT-FADD for 3–12 h, the TAT-FADD was immunoprecipated (IP) using anti-His antibody followed by analysis of DISC assembly proteins procaspase-8 and cFLIP$_L$, molecular weight marker

left to each blot. (**B**) Representative flow quadrants of Figure 3B. Treatment of 5 μM of TAT-FADD for 3–12 h and expression of cFLIPL in (**C**) MCF-7 and (**D**) HeLa cells. (**E**–**G**) HCT 116 cells were left untreated and pre-treated with zVAD (+zVAD), followed by treatment of 5 μM TAT-FADD for 3–12 h, (**E**) Cells were stained with Annexin-PI staining kit, and analyzed by fluorescent microscoe, representative of 150 cells from 3 different fields; scale bar 5 μm and (**F**) % cell viability and, (**G**) apoptotic death by Tali™ image based cytometer, control represents non-treated (NT) cells at 0h. (H-L) HCT 116 cells were transfected with pcDNA3-cFLIPL for 48h (lane 2 & 5), primed with TNFα (10 ng/mL) for 12 h (lane 3 & 6) followed by treatment with 5 μM of TAT-FADD for 6 h (lane 4, 5 & 6), (**H**) Cells were stained with Annexin-PI staining kit, and analyzed by fluorescent microscoe, representative of 150 cells from 3 different fields; scale bar 5 μm and (**I**) % cell viability, (**J**) % change in MMP, (**K**) measurement of caspase-8 activity and (**L**) measurement of caspase-3 activity. Control represents a vector transfected and non TNFα primed cells. In **F**, **G** significance compared between non-treated (for zVAD and TAT-FADD, white bars) and TAT-FADD treated cells. In **I**–**L** significance compared between non-treated (white bars) and TAT-FADD treated cells (black bars), control in white bar represents a vector transfected and non TNFα primed cells non-treated (white bars) and TAT-FADD treated cells (black bars), control in white bar represents a vector transfected and non TNFα primed cells. h, hours; PI, propidium iodide; MMP, mitochondrial membrane potential. Mean ± SD; $^{**}p \le 0.01$ and $^{***}p \le 0.001$.

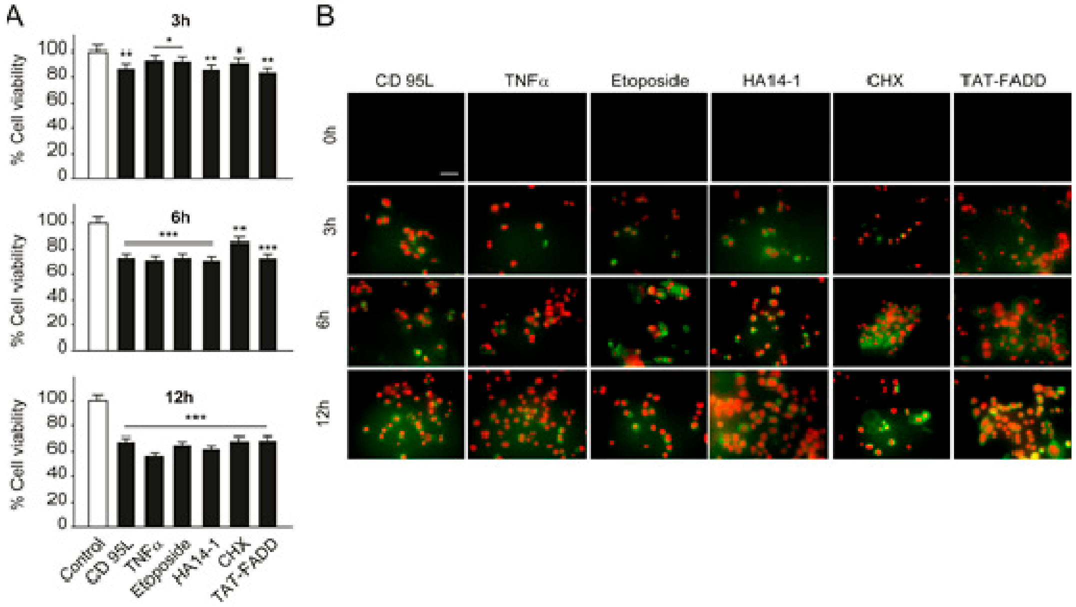

Figure S4. TAT-FADD comparision with conventional apoptosis inducers. (**A**–**B**) HCT 116 cells were treated with CD 95L (200 ng/mL), TNF-α (50 ng/mL), etoposide (50 μM), HA14-1 (5 μM), protein translational inhibitor cycloheximide (CHX, 5 μg/mL) and TAT-FADD (5 μM) alone for the mentioned time points, (**A**) % cell viability and (**B**) Cells were stained with Annexin-PI staining kit, and analyzed by fluorescent microscoe, representative of 150 cells from 3 different fields; scale bar 5 μm. In **A** significance compared between non-treated (white bars) and TAT-FADD treated cells (black bars). h, hours. Mean ± SD; $^{*}p \le 0.05$; $^{**}p \le 0.01$ and $^{***}p \le 0.001$.

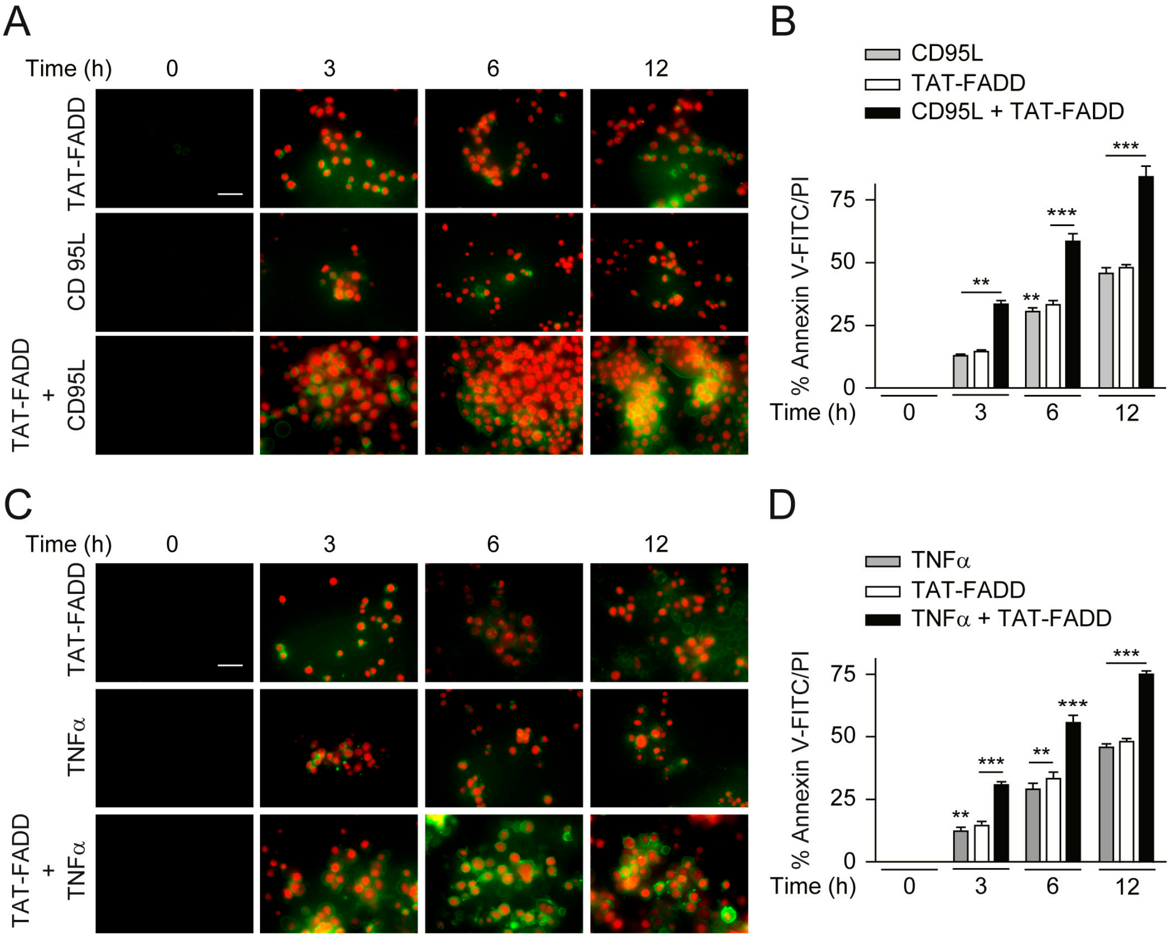

Figure S5. TAT-FADD synergestically enhances death ligands apoptotic competency. (**A**–**B**) HCT116 cells were treated alone with TAT-FADD (5 μM), CD 95L (200 ng/mL) and in combination for 3–12 h, (**A**) Cells were stained with Annexin-PI staining kit, and analyzed by fluorescent microscoe, representative of 150 cells from 3 different fields; scale bar 5 μm and (**B**) % apoptotic death by TaliTM image based cytometer. (**C**–**D**) HCT116 cells were treated alone with TAT-FADD (5 μM), TNF-*α* (50 ng/mL) and in combination for 3–12 h, (**C**) Cells were stained with Annexin-PI staining kit, and analyzed by fluorescent microscoe, representative of 150 cells from 3 different fields; scale bar 5 μm and (**D**) % apoptotic death by TaliTM image based cytometer. Control represents untreated cells (0 h). In **B**, **D** significance compared between non-treated at 0 h and treated cells. Mean ± SD; $^{**}p \le 0.01$ and $^{***}p \le 0.001$.

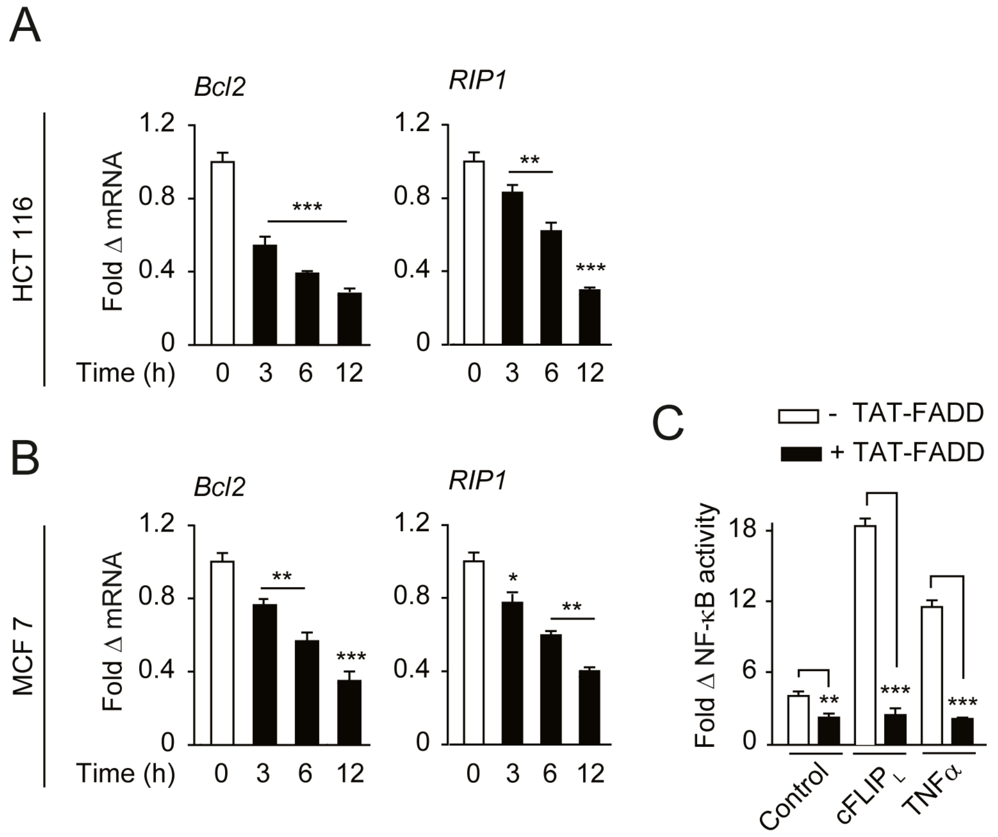

Figure S6. TAT-FADD suppresses NF-κB signaling downstream genes in cancer cells. Cells were treated with 5 μM of TAT-FADD for 3–12 h and mRNA expression of *Bcl2* and *RIP1* (**A**) in HCT 116 cells and (**B**) in MCF-7 cells. The 0 h represents untreated cells taken as controls. (**C**) HCT 116 cells were transfected with pcDNA3-cFLIP$_L$ for 48 h (lane 2 & 3), primed with TNFα (10 ng/mL; lane 5 & 6) for 12 h, followed by treatment with 5 μM of TAT-FADD for 6 h (lane 2, 4 & 6) and measurement of NF-κB luciferase activity. In **A** & **B** significance compared between non-treated (represents as 0 h) and TAT-FADD treated cells. In **C** significance compared between non-treated (white bars) and TAT-FADD treated cells (black bars), control in white bar represents a vector transfected and non TNFα primed cells. h, hours. Mean ± SD; $^{*}p \le 0.05$, $^{**}p \le 0.01$ and $^{***}p \le 0.001$.

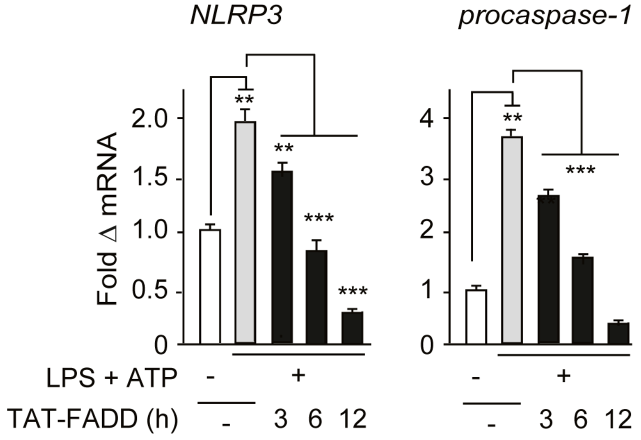

Figure S7. TAT-FADD suppresses NLRP3 inflammasome primed activation. HCT116 cells were primed with LPS (100 ng/mL, 12 h) and ATP (5 mM, 2 h) followed by treatment with 5 μM of TAT-FADD for 3–12 h and mRNA expression of *NLRP3* and *procaspase-1*. The white bar represents un-primed and non-treated cells for 12 h and taken as controls. Representative of 3 independent

experiments in triplicates. Significance is compared between non-treated (white bars) and LPS with ATP primed cells (gray bars); and between LPS with ATP primed cells and TAT-FADD treated cells (black & dark gray bars respectively). h, hours. Mean ± SD; $^{**}p \leq 0.01$ and $^{***}p \leq 0.001$.

Supplementary Table S1. List of antibodies used in the study.

ANTIBODIES	COMPANY	CATLOUGE
Rabbit TRAF2 (C192)	Cell Signaling Technology	4724
Rabbit NF-κB p65 (D14E12)	Cell Signaling Technology	8242
Rabbit phospho-NF-κB p65 (Ser536) (93H1)	Cell Signaling Technology	3033
Mouse IκBα (L35A5)	Cell Signaling Technology	4814
Rabbit Phospho-IκBα (Ser32) (14D4)	Cell Signaling Technology	2859
Rabbit IKKβ (D30C6)	Cell Signaling Technology	8943
Mouse p53 (1C12)	Cell Signaling Technology	2524
Rabbit caspase-9	Cell Signaling Technology	9502
Rabbit caspase-7	Cell Signaling Technology	9492
Rabbit cytochrome c	Cell Signaling Technology	4272
Rabbit PARP	Cell Signaling Technology	9542
Rabbit RIP (D94C12)	Cell Signaling Technology	3493
Rabbit caspase-1	Cell Signaling Technology	2225
Rabbit IL-1β (D3U3E)	Cell Signaling Technology	12703
Rabbit NLRP3 (D2P5E)	Cell Signaling Technology	13158
Rabbit β-Actin	Cell Signaling Technology	4967
Anti-rabbit IgG, HRP-linked	Cell Signaling Technology	7074
Rabbit FLIP	Novus Biologicals	NBP1-45479
Mouse Bcl-2 (Bcl-2-100)	Thermofisher Scientific	13-8800
Mouse Anti-Human Caspase-8	BD Pharmigen	551242
Mouse Anti-Human c-IAP-2	BD Pharmigen	552782
Ubiquitin monoclonal antibody (P4D1)	Enzo Life Sciences, Inc.	BML-PW093

Supplementary Table S2. List of real time PCR primers used in the study.

GENE	PRIMER SEQUENCE
cFLIP$_L$	Fwd: 5' TGGCCTCCCTCAAGTTCCT 3'<br>Rev: 5' TGGAATAACATCAAGGCATCCTT 3'
cIAP2	Fwd: 5' TCCAAGGTGTGAGTACTTGATAAGAATT 3'<br>Rev: 5' CTGATGTGGATAGCAGCTGTTCA 3'
Bcl-2	Fwd: 5' TGCGGCCTCTGTTTGATTTC 3'<br>Rev: 5' GGGCCAAACTGAGCAGAGTCT 3'
RIP1	Fwd: 5' AATGGCGGCACCCTCTACTA 3'<br>Rev: 5' TCCGACTTCTCTGTGGGCTTT 3'
NLRP3	Fwd: 5' TGCCCCGACCCAAACC 3'<br>Rev: 5' GAAGCCGTCCATGAGGAAGA 3'
Procaspase-1	Fwd: 5' ATACCAAGAACTGCCCAAGTTTG 3'<br>Rev: 5' GGCAGGCCTGGATGATGA 3'
Pro IL-1β	Fwd: 5' GACAACGAGGCGTACGTTCA 3'<br>Rev: 5' CGATTTCTGTTGACTATCCCGTAA 3'
18S rRNA	Fwd: 5' AGAAACGGCTACCACATCCAA 3'<br>Rev: 5' TGTCACTACCTCCCCGTGTCA 3'
